# Tumor vessel phenotype in colorectal cancer microenvironment according to age at diagnosis

**DOI:** 10.1038/s41416-026-03373-6

**Published:** 2026-03-25

**Authors:** Kosuke Matsuda, Satoko Ugai, Satoshi Miyahara, Qian Yao, Jules Cazaubiel, Nobuhiro Nakazawa, Mayu Higashioka, Yuxue Zhong, Andrew T. Chan, Jeffrey A. Meyerhardt, Kimmie Ng, Mingyang Song, Juha P. Väyrynen, Jonathan A. Nowak, Marios Giannakis, Tomotaka Ugai, Shuji Ogino

**Affiliations:** 1 Program in MPE Molecular Pathological Epidemiology, Department of Pathology, Brigham and Women’s Hospital, and Harvard Medical School, Boston, MA USA; 2 Department of Epidemiology, Harvard T.H. Chan School of Public Health, Boston, MA USA; 3 Department of Medical Oncology, Dana-Farber Cancer Institute, Boston, MA USA; 4 Clinical and Translational Epidemiology Unit, Massachusetts General Hospital and Harvard Medical School, Boston, MA USA; 5 Division of Gastroenterology, Massachusetts General Hospital and Harvard Medical School, Boston, MA USA; 6 Department of Nutrition, Harvard T.H. Chan School of Public Health, Boston, MA USA; 7 Translational Medicine Research Unit, University of Oulu, Medical Research Center Oulu, and Oulu University Hospital, Oulu, Finland; 8 Broad Institute of MIT and Harvard, Cambridge, MA USA; 9 Division of Integrative Cancer Research, National Cancer Center Research Institute, Tokyo, Japan; 10 Department of Medicine, Brigham and Women’s Hospital and Harvard Medical School, Boston, MA USA; 11 Institute of Science Tokyo, Tokyo, Japan

**Keywords:** Cancer microenvironment, Colorectal cancer, Tumour angiogenesis

## Abstract

**Background:**

Given the global issue of the rising incidence of early-onset colorectal cancer (CRC), we tested the hypothesis that tumor vasculature phenotypes might vary with age at CRC diagnosis.

**Method:**

We used in situ multispectral immunofluorescence combined with digital image analysis and machine learning to measure expression of endothelial cell markers [ACKR1 (DARC), CD34, CD36, KDR (VEGFR2), LAMB1 (laminin β1), MADCAM1] and KRT (keratin) in 843 tumors derived from 4476 CRC cases in U.S.-wide prospective cohorts under the prospective cohort incident-tumor biobank method.

**Results:**

Overall CD34^+^ vessel and CD34^+^LAMB1^+^ vessel densities inversely correlated with younger age at CRC diagnosis (both *P*_trend_ < 0.0001). In the inverse probability-weighted multivariable-adjusted logistic regression analyses, compared to age ≥70, odds ratios (with 95% confidence interval) for high (vs. low) overall vessel density were 0.85 (0.74–0.99) for age 55–69 and 0.63 (0.48–0.81) for age <55, and those for high (vs. low/negative) CD34^+^LAMB1^+^ vessel density were 0.56 (0.47–0.65) for age 55–69 and 0.28 (0.20–0.40) for age <55.

**Conclusions:**

Hypovascularities of overall and CD34^+^LAMB1^+^ vessels may be microenvironmental characteristics of early-onset CRC if validated by independent studies. Our findings highlight age-related tumor pathobiological differences. Identifying specific biomarkers of early-onset CRC can provide pathogenetic and etiological clues.

## Introduction

Tumor vasculature is an essential component of the tumor microenvironment. While tumor vessels supply oxygen and nutrients that promote tumor growth, they can also play a role in antitumor immunity [1]. Recent single-cell RNA sequencing analyses have revealed substantial heterogeneity among vascular endothelial cells in the tumor microenvironment [2–4]. Cellular phenotyping and quantitative morphological evaluations of tumor vasculature can provide valuable insights into its role in the tumorigenic process.

The age of colorectal cancer (CRC) diagnosis receives increasing attention as the incidence of early-onset CRC, commonly defined as CRC diagnosed in adults under age 50, has been steadily rising for unclear reasons [5–11]. Early-onset CRC appears to exhibit different tumor immune characteristics and stromal cell compositions compared to later-onset CRC [12–14]. In addition to age-related changes in tissue characteristics, tumor-host interactions may contribute to phenotypes of the tumor microenvironment, including vasculature, during the carcinogenic process [15–17]. However, to our knowledge, no prior studies have examined detailed tumor vascular phenotypes in relation to age at CRC diagnosis.

Therefore, we tested the hypothesis that tumor vessel phenotypes and distribution might vary with the age at diagnosis, using an in-situ multispectral immunofluorescence assay combined with computational image analysis and machine learning on incident CRC cases within two U.S.-wide prospective cohort studies.

## Methods

### Study population

We used the prospective cohort incident-tumor biobank method (PCIBM) [18–20] and two U.S.-wide prospective cohort studies: the Nurses’ Health Study (NHS), enrolling 121,700 women aged 30 to 55 years in 1976, and the Health Professionals Follow-up Study (HPFS; NCT00005182), enrolling 51,529 men aged 40 to 75 years in 1986 [21]. Every two years, follow-up questionnaires had been sent to cohort participants to identify newly diagnosed cancers in themselves and their first-degree relatives. The response rate exceeded 90% for each follow-up questionnaire cycle in both cohorts. The National Death Index was used to ascertain death and identify participants with unreported lethal colorectal cancer (CRC). For physical activity, the metabolic equivalent of task score (METS) per hour was calculated based on intensity and duration, and then it was quantified as the weekly sum of all activities (METS hour/week). Alcohol consumption was computed by summing ethanol intake from beer, wine, and liquor. We used cumulative average values for those factors using all available questionnaires before CRC diagnosis.

We successfully identified a total of 4476 incident CRC cases that had occurred during follow-up of the 173,229 participants. Study physicians, blinded to exposure data, reviewed documentation of identified CRC cases to confirm the diagnosis of colorectal adenocarcinoma (for which the common term CRC is used in this study) and record clinical and tumor characteristics.

**Fig. 1:**
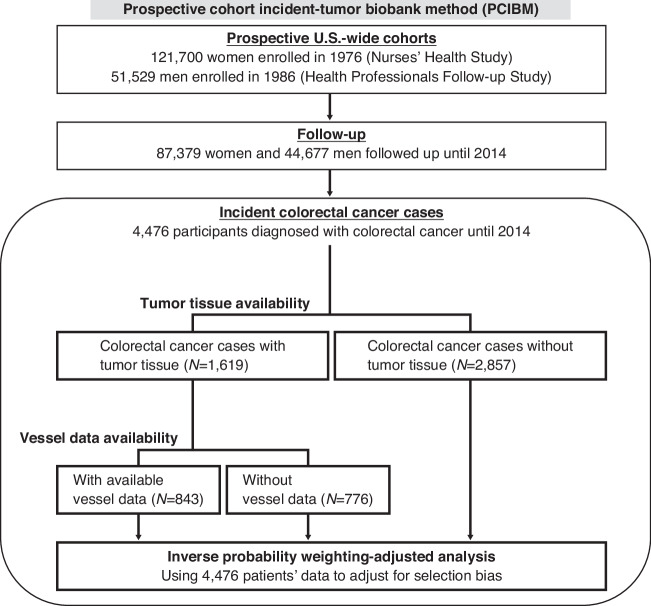
Flowchart of the study population. The prospective cohort incident-tumor biobank method (PCIBM) encompassing the entirety of the above elements enabled us to collect incident-tumor tissue specimens, conduct multivariable-adjusted analyses, and use the inverse probability weighting method to adjust for selection bias due to tissue data availability. These features increase the generalizability of findings.

Formalin-fixed, paraffin-embedded (FFPE) tissue blocks had been collected since 1997 for the HPFS cohort and since 2001 for the NHS cohort from study participants with CRC who had undergone tumor resection at hospitals across the U.S. [22]. Hematoxylin and eosin-stained sections were reviewed by S.O. to evaluate tumor differentiation and lymphocytic reaction patterns. Tissue microarrays were constructed from a subset of tumors, comprising multiple 0.6 mm cores per tumor. Similarly, non-tumorous tissue was collected and placed on the same tissue microarray blocks as the tumor tissue. As detailed below, a multispectral immunofluorescence assay was performed on tissue microarray slides, yielding valid assay data for 843 CRC cases. Nonetheless, we leveraged available data of the 4476 incident colorectal cancer cases (that had occurred among the 173,229 participants) to adjust for selection bias due to the tissue data availability (Fig. 1).

The study protocol was approved by the institutional review boards of the Brigham and Women’s Hospital and Harvard T.H. Chan School of Public Health (IRB protocol number: 2019P003588), and those of participating registries as required. All clinicopathological and molecular analyses were performed while blinded to other data. Informed consent was obtained from all study participants.

### Tumor molecular analyses

DNA was extracted from tumor areas in whole tissue sections using the QIAamp DNA Mini Kit (Qiagen, Valencia, CA), and the following analyses were conducted: tumor microsatellite instability (MSI) status (based on ten microsatellites: D2S123, D5S346, D17S250, BAT25, BAT26, BAT40, D18S55, D18S56, D18S67, and D18S487); CpG island methylator phenotype (CIMP) status (based on eight CIMP-specific promoters: *CACNA1G*, *CDKN2A*, *CRABP1*, *IGF2*, *MLH1*, *NEUROG1*, *RUNX3*, and *SOCS1*); long interspersed nucleotide element-1 (LINE-1) methylation level; mutation statuses of *KRAS* (codons 12, 13, 61, and 146), *BRAF* (codon 600), and *PIK3CA* (exons 9 and 20), as previously described [21, 23–25].

### Multispectral immunofluorescence

**Fig. 2:**
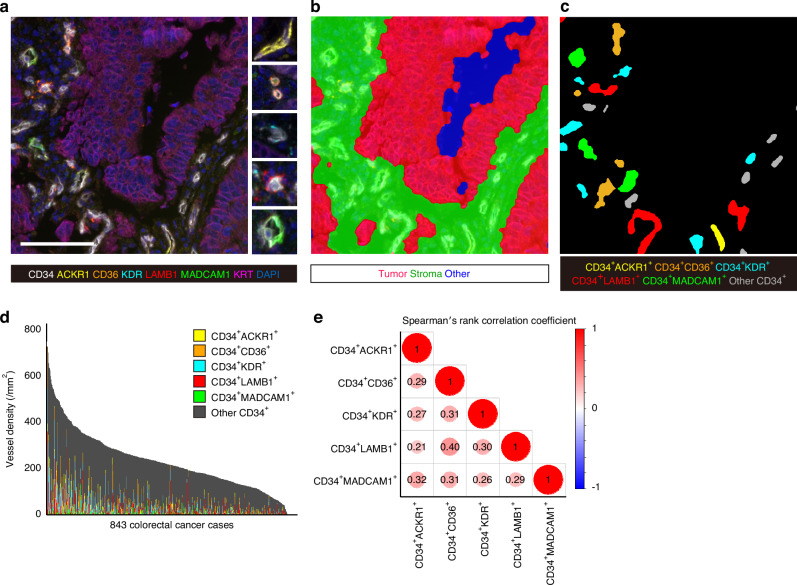
Molecular phenotyping of vessels using multispectral immunofluorescence. **a** A representative multispectral immunofluorescence image for detection of ACKR1, CD34, CD36, KDR, LAMB1, MADCAM1, KRT, and DAPI (nuclear stain). Scale bar: 100 μm. **b** The segmentation processed image to identify tumor epithelial and stromal regions. **c** The processed image to classify vessels by predominant markers. **d** The distribution of vessel densities in the 843 colorectal cancer cases. **e** A matrix plot of Spearman’s rank correlation coefficients among the marker-positive vessel densities.

To profile tumor vasculature, we designed a custom multispectral immunofluorescence assay based on the tyramide signal amplification method (Supplementary Table S1, Supplementary Fig. S1). Antibodies to visualize specific endothelial cells were selected based on data from the literature and the Human Protein Atlas (https://www.proteinatlas.org/) [2, 3, 26–28]. We then constructed a multispectral immunofluorescence assay targeting endothelial cells, including ACKR1 (HGNC:4035; atypical chemokine receptor 1; DARC; venous marker), CD34 (HGNC:1662; endothelial cell marker), CD36 (HGNC:1663; stalk-like cell marker), KDR (HGNC:6307; kinase insert domain receptor; VEGFR2; tip^SII-III^-like cell marker), LAMB1 (HGNC:6486; laminin β1; tip^SI^-like cell marker), MADCAM1 (HGNC:6765; mucosal vascular addressin cell adhesion molecule 1; high-endothelial venule marker), KRT (keratin; epithelial cell marker), according to standardized nomenclature recommended by the expert panel, [29] as well as DAPI (4′,6-diamidino-2-phenylindole; nuclear marker) (Fig. 2a). We optimized the combination of antibodies and fluorochromes, their concentrations, and the order of staining, and confirmed the correspondence between the multispectral immunofluorescence staining pattern and those of single-marker chromogenic immunohistochemistry (Supplementary Fig. S2). The Opal 6-Plex Manual Detection Kit (Cat#. NEL811001KT, Akoya Biosciences, Hopkinton, MA, USA) was used for staining, and PhenoImager HT 2.0 (Akoya Biosciences, RRID: SCR_023772) was used for immunofluorescence observation and imaging.

### Image acquisition and analysis

The immunofluorescent slides were scanned using PhenoImager HT 2.0 which is equipped with seven imaging filters and 20x objective lens. Cores with non-tumorous mucosa, marked necrosis, or extremely few tumor cells were excluded from the analysis. The acquired images were processed using inForm Tissue Analysis Software version 3.0.0 (Akoya Biosciences, RRID: SCR_019155) for autofluorescence adjustment and spectral unmixing. The unmixed images were 1600 × 1600 pixels (0.5 × 0.5 µm^2^/pixel) in size and underwent preprocessing in a standardized pipeline built with MATLAB software R2023b (MathWorks, Natick, MA, USA, RRID: SCR_001622). Noise was reduced using a 9 × 9 averaging filter to smooth pixel-level fluctuations while preserving overall structural features. Image binarization was performed using Otsu’s thresholding method to separate signal from background. To refine object contours and eliminate small artifacts, morphological operations were applied using a circular structuring element, specifically closing followed by opening. The processed binary images were then reviewed in conjunction with the original fluorescent images by a pathologist (K.M.), who manually excluded any images or regions with staining artifacts, segmentation errors, or inadequate signal quality. Signal intensity stability and adequate control of potential fluorescent leakage were validated through appropriate experimental procedures (Supplementary Figs. S3–4). The current study used 1444 core images for 843 cases (average 1.7 cores). The area of each core was calculated from images containing all spectra, while the epithelial area was determined from the Opal 780 (KRT) images. The stromal area was calculated by subtracting the epithelial area from the core area (Fig. 2b). From the Opal 690 (CD34) images, the coordinates and morphological features of CD34^+^ vessels were extracted. The positivity of other endothelial cell markers for each CD34^+^ vessel was also obtained, and markers were considered positive if they occupied more than 5% of the vessel area (Fig. 2c).

### Morphological classification of vessels

**Fig. 3:**
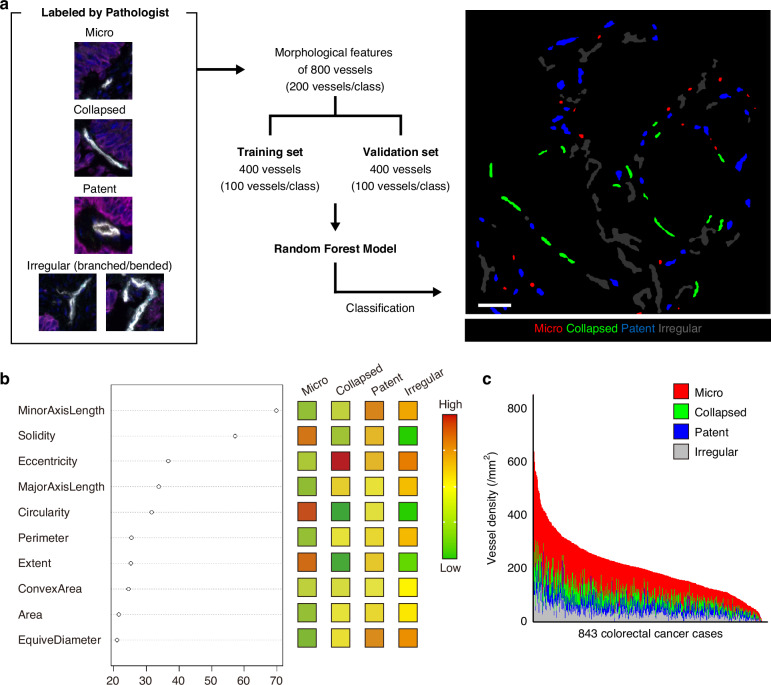
Morphological classification of vessels using a pathologist-supervised random forest model. **a** CD34^+^ vessels were classified into the following four categories; micro: microvessel with an indistinct lumen; collapsed: vessel with an obstructed lumen due to compression from the surrounding tissue; patent: vessel with a preserved lumen; irregular: vessel not classified above, sometimes complex, bent, and branched. Scale bar: 100 μm. **b** Variable importance plots in the developed random forest model to classify vessels by morphological features. Each feature was standardized by z-score, and the median value of each vessel class is indicated by the color. **c** The distribution of densities of morphologically classified vessels in the 843 colorectal cancer cases.

Individual images of each CD34^+^ vessel were cropped from tissue images containing Opal 690 (CD34), Opal 780 (KRT), and DAPI. From these vessel images, automatically selected ones were labeled by a pathologist (K.M.) on at least 200 vessels each in the following four categories based on morphology; “micro” (microvessel with an indistinct lumen); “collapsed” (vessel with an obstructed lumen due to compression from the surrounding tissue); “patent” (vessel with a preserved lumen); “irregular” (complex, bended, and/or branched vessel not classified above). A second pathologist (S.M.) independently labeled 100 of these vessels, and interobserver reproducibility was evaluated (concordance rate = 91%, and unweighted Cohen’s κ = 0.88). The training set consisted of 400 vessels randomly selected from the labeled images, 100 in each category, and the validation set consisted of another 400 vessels randomly selected from the remaining images, 100 in each category. A random forest model to classify vessels was developed using randomForest package (RRID: SCR_015718) in R software version 4.4.0 (R Foundation for Statistical Computing, Vienna, Austria, RRID:SCR_001905). Based on the morphological features (Area, Circularity, ConvexArea, Eccentricity, EquivDiameter, Extent, MajorAxisLength, MinorAxisLength, Perimeter, and Solidity [30]) of the training set and validated with the validation set (Fig. 3a), the random forest classifier was constructed using 1000 decision trees, with four features randomly selected as candidates at each node split.

### Statistical analysis

Statistical analyses were performed using SAS version 9.4 (SAS Institute, Cary, NC, USA, RRID: SCR_008567). R software was used for data visualization. All *p*-values were two-sided. We used the stringent two-sided α level of 0.005 (≈ 0.05/9), adjusting for the 9 vessel variables. Our primary hypothesis testing was an assessment of the association of age at diagnosis with vessel densities. All other assessments were secondary analyses. The Spearman correlation test was performed to assess the correlations of age (continuous) with each of the continuous vessel metrics (and covariates in secondary analyses). To adjust for potential confounding, we conducted multivariable-adjusted logistic regression analyses that assessed the relationship of age with overall CD34^+^ vessel density (low vs. high), CD34^+^LAMB1^+^ vessel density (negative/low vs. high), and micro CD34^+^ vessel density (low vs. high) as outcome variables. These vessel metrics did not follow the normal distribution. Overall CD34^+^ vessel and micro CD34^+^ vessel density variables were dichotomized (low vs. high) using each median value as a cutpoint. CD34^+^LAMB1^+^ vessel density was dichotomized into high (above the median among cases with non-zero values) and low/negative. We initially included the following covariates: sex (female vs. male), body mass index (<30 vs. ≥30 kg/m^2^), pack-years of smoking (0 vs. 1–39 vs. ≥40), family history of colorectal cancer in any first-degree relative (present vs. absent), tumor location (proximal colon vs. distal colon vs. rectum), MSI status (non-MSI-high vs. MSI-high), CIMP status (negative/low vs. high), LINE-1 methylation level (≤55% vs. 55–65% vs. >65%), *KRAS* mutation (mutated vs. wild-type), *BRAF* mutation (mutated vs. wild-type), and *PIK3CA* mutation (mutated vs. wild-type). A backward elimination was conducted with a threshold *p*-value of 0.1 to select variables for the final model.

Cases with missing data [family history of CRC (0.5%), tumor location (0.4%), MSI (3.1%), CIMP (7.6%), *KRAS* (3.0%), *BRAF* (2.3%), and *PIK3CA* mutation (8.8%)] were included in the majority category of a given categorical covariate to limit the degrees of freedom of the models, and missing indicator variables were assigned to cases with missing data in the relevant variables. For the cases with missing data on LINE-1 methylation (2.9%), we assigned a separate indicator variable. To adjust for selection bias in the CRC cases due to tissue data availability, the inverse probability weighting (IPW) method using all of the incident 4476 CRC cases was integrated into multivariable-adjusted logistic regression models, as previously described [31].

## Results

### Multispectral immunofluorescence assay to evaluate tumor vessel density

Multispectral immunofluorescence staining to assess vessel density and morphology was performed across 10 tissue microarray sections, yielding data on 61,550 vessels from 843 colorectal cancers (CRCs), which were among 4476 CRC cases that had occurred in the two prospective cohort studies (Fig. 2a, b). Marker intensities were consistent across the sections (Supplementary Fig. S4). Among endothelial cells comprising CD34^+^ vessels, ACKR1 (DARC), CD36, KDR (VEGFR2), LAMB1 (laminin β1), and MADCAM1 were expressed in the cell membrane or cytoplasm. ACKR1 expression was also observed in non-vascular stromal cells, while LAMB1 was detected in a small number of tumor epithelial cells. Each vessel was composed of multiple endothelial cells, and CD36, KDR, and LAMB1 were often expressed in only a subset of these cells, rather than uniformly across the entire vessel. When expression of a given marker exceeded 5% of the vessel area, that vessel was called “positive for the marker”. Thus, vessels were profiled by the predominant marker (Fig. 2c). The proportions of vessels positive for each marker in the 61,550 CD34^+^ vessels were 4.2% for ACKR1, 6.7% for CD36, 14% for KDR, 6.8% for LAMB1, and 1.4% for MADCAM1 (Fig. 2d, Supplementary Fig. S5). Densities of subtypes of vessels in each tumor showed low to moderate correlation (Fig. 2e).

### Morphological classification of vessels

A random forest model was developed to classify tumor vessels into four categories (micro, collapsed, patent, and irregular) based on morphological features extracted from the vessels, and the model achieved 96% accuracy in the training set and 92% in the validation set (Fig. 3a, Supplementary Tables S2-3). Common misclassifications by the classifier were the classification of collapsed or patent vessels as irregular vessels. The morphological features of the classified vessels and their variable importance are shown in Fig. 3b. Micro CD34^+^ vessels were characterized by lower area and minor axis length, along with higher circularity, extent, and solidity, reflecting smaller and rounder morphology. Collapsed CD34^+^ vessels showed lower minor axis length and higher eccentricity, consistent with their elongated appearance. Patent CD34^+^ vessels had higher minor axis length and circularity, reflecting their larger and rounder form. Irregular CD34^+^ vessels exhibited higher area and lower circularity, extent, and solidity, consistent with their larger and irregular shape. The proportions of the morphologically classified vessels in the 61,550 vessels were 18% for collapsed, 49% for micro, 14% for patent, and 18% for irregular (Fig. 3c).

### Tumor vessel features by age at diagnosis

**Fig. 4:**
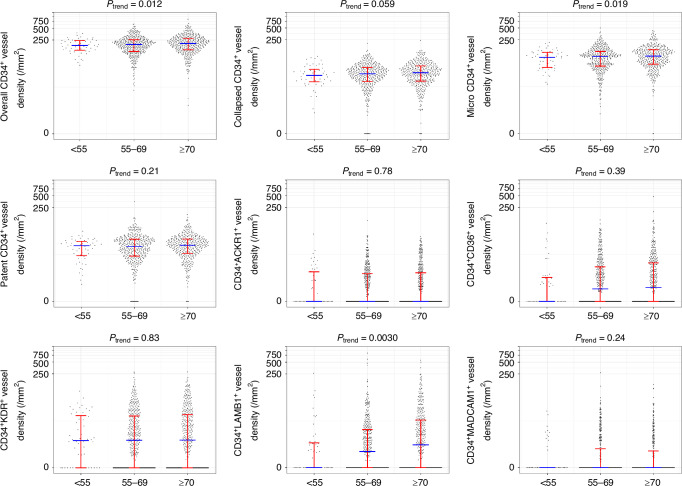
Distribution of each vessel density in different age groups. In the scatter dot plot, the blue horizontal bar indicates the median, with the red horizontal bars marking the interquartile range (25th–75th percentile). The Spearman’s rank correlation test was performed on continuous age and vessel density data to calculate *P*_trend_. The vertical axes are pseudo-log transformed.

Among the 843 CRC cases with available tumor vessel data, there were 14 early-onset cases diagnosed before age 50 years, 38 cases diagnosed at age 50-54, 400 cases diagnosed at age 55–69, and 391 cases diagnosed at or after age 70. While we used a combined category of cases under age 55 because of the small number of cases under age 50, many of our statistical analyses used the continuous age variable [Table 1; features according to the four age groups (<50, 50–54, 55–69, ≥70) in Supplementary Table S4]. The Spearman test showed the correlations of age (continuous) with the densities of CD34^+^LAMB1^+^ vessels (*P* = 0.0030), overall CD34^+^ vessels (*P* = 0.012, insignificant at the adjusted alpha level of 0.005), and micro vessels (*P* = 0.019) (Fig. 4), but not vessels with other phenotypes. There was no significant correlation between age at diagnosis and overall CD34^+^ vessel density in tumor-adjacent normal mucosa (*P* = 0.66) (Supplementary Fig. S6). Considering the associations of MSI-high status with both age and tumoral microenvironmental features, we examined clinicopathological and vessel features in non-MSI-high stratum (Supplementary Table S5) [with MSI-high stratum data not shown due to low case count (*n* = 5) of MSI-high CRC under 55]. Among non-MSI-high tumors, the Spearman test showed the correlations of age with the densities of CD34^+^LAMB1^+^ vessels (*P* = 0.012) and overall CD34^+^ vessels (*P* = 0.043).

**Table 1:** Clinical, pathological, and molecular characteristics of colorectal cancer cases according to age at diagnosis.

		Age at diagnosis			
Characteristics^a^	All cases	<55	55–69	≥70	*P* value^b^
	*N*=843	*N*=52	*N*=400	*N*=391	
Sex					<0.0001
Female (NHS)	468 (56%)	40 (77%)	249 (62%)	179 (46%)	
Male (HPFS)	375 (44%)	12 (23%)	151 (38%)	212 (54%)	
Body mass index preceding diagnosis (kg/m^2^)	26 (24–29)	26 (24–29)	26 (24–29)	25 (23–28)	0.0022
Prediagnostic physical activity (METS hours/week)	13 (3.7–29)	11 (6.0–17)	12 (3.6–31)	13 (3.8–29)	0.85
Prediagnostic alcohol consumption (g/day)	2.3 (0–12)	1.9 (0–7.7)	2.4 (0–11)	2.2 (0–13)	0.83
Prediagnostic pack-year of smoking	7.0 (0–30)	0 (0–13)	7.5 (0–31)	10 (0–33)	0.11
Family history of colorectal cancer in a first-degree relative					0.42
Absent	659 (79%)	39 (76%)	315 (79%)	305 (79%)	
Present	176 (21%)	12 (24%)	82 (21%)	82 (21%)	
Tumor location					0.0034
Proximal colon	420 (50%)	19 (37%)	184 (46%)	217 (56%)	
Distal colon	248 (30%)	21 (40%)	132 (33%)	95 (24%)	
Rectum	172 (20%)	12 (23%)	83 (21%)	77 (20%)	
AJCC disease stage					0.0003
I	183 (23%)	11 (22%)	74 (20%)	98 (27%)	
II	254 (33%)	10 (20%)	123 (33%)	121 (34%)	
III	226 (29%)	22 (44%)	110 (29%)	94 (26%)	
IV	119 (15%)	7 (14%)	67 (18%)	45 (13%)	
Tumor differentiation					0.86
Well/ Moderate	773 (92%)	48 (92%)	369 (92%)	356 (91%)	
Poor	69 (8%)	4 (8%)	31 (8%)	34 (9%)	
MSI status					0.055
Non-MSI-high	674 (82%)	46 (90%)	328 (84%)	300 (80%)	
MSI-high	143 (18%)	5 (10%)	62 (16%)	76 (20%)	
CIMP status					0.0006^c^
Negative/ Low	633 (81%)	47 (90%)	322 (85%)	264 (76%)	
High	146 (19%)	5 (10%)	57 (15%)	84 (24%)	
LINE-1 methylation level					0.0009^d^
≤55%	180 (22%)	13 (26%)	88 (22%)	79 (20%)	
55–65%	329 (39%)	21 (41%)	167 (42%)	141 (37%)	
>65%	325 (39%)	17 (33%)	142 (36%)	166 (43%)	
*KRAS* mutation					0.51
Wild type	490 (60%)	37 (74%)	229 (59%)	224 (59%)	
Mutated	328 (40%)	13 (26%)	160 (41%)	155 (41%)	
*BRAF* mutation					0.49
Wild type	692 (84%)	43 (84%)	330 (84%)	319 (84%)	
Mutated	132 (16%)	8 (16%)	62 (16%)	62 (16%)	
*PIK3CA* mutation					0.21
Wild type	641 (83%)	45 (92%)	292 (83%)	304 (83%)	
Mutated	127 (17%)	4 (8%)	61 (17%)	62 (17%)	
Tumor-infiltrating lymphocytes					0.028
Absent/ Low	606 (73%)	42 (80%)	291 (74%)	273 (70%)	
Intermediate	132 (16%)	6 (12%)	58 (15%)	68 (17%)	
High	97 (12%)	4 (8%)	44 (11%)	49 (13%)	
Intratumoral periglandular reaction					0.98
Absent/ Low	110 (13%)	5 (10%)	43 (7%)	62 (16%)	
Intermediate	622 (74%)	44 (85%)	307 (49%)	271 (69%)	
High	104 (12%)	3 (6%)	271 (44%)	57 (15%)	
Peritumoral lymphocytic reaction					0.95
Absent/ Low	128 (15%)	5 (10%)	44 (11%)	79 (20%)	
Intermediate	574 (69%)	42 (80%)	303 (77%)	229 (59%)	
High	131 (16%)	5 (10%)	45 (11%)	81 (21%)	
Crohn's-like lymphoid reaction					0.15
Absent/ Low	522 (74%)	29 (83%)	249 (75%)	244 (72%)	
Intermediate	126 (18%)	3 (9%)	60 (18%)	63 (19%)	
High	56 (8%)	3 (9%)	23 (7%)	30 (9%)	
Vessel density (/mm^2^)					
Overall CD34^+^	197 (133–262)	182 (134–241)	191 (127–254)	205 (140–277)	0.012
Collapsed CD34^+^	34 (21–51)	31 (21–49)	33 (21–48)	36 (22–54)	0.059
Micro CD34^+^	93 (56–134)	92 (49–122)	92 (52–126)	95 (59–139)	0.019
Patent CD34^+^	27 (15–39)	27 (15–35)	25 (14–39)	27 (17–39)	0.21
CD34^+^ACKR1^+^	0 (0–5.2)	0 (0–5.5)	0 (0–4.9)	0 (0–5.4)	0.78
CD34^+^CD36^+^	1.7 (0–8.5)	0 (0–4.0)	1.6 (0–7.5)	1.7 (0–9.5)	0.39
CD34^+^KDR^+^	5.0 (0–22)	4.7 (0–22)	5.0 (0–21)	5.0 (0–23)	0.83
CD34^+^LAMB1^+^	2.4 (0–12)	0 (0–4.2)	2.1 (0–9.0)	3.5 (0–16)	0.0030
CD34^+^MADCAM1^+^	0 (0–2.3)	0 (0–0)	0 (0–2.6)	0 (0–2.3)	0.24

*AJCC* American Joint Committee on Cancer, *CIMP* CpG island methylator phenotype, *HPFS* Health Professionals Follow-up Study, *LINE-1* long interspersed nucleotide element-1, *METS* metabolic equivalent of task score, *MSI* microsatellite instability, *NHS* Nurses’ Health Study.

^a^Percentage indicates the proportion of patients with a specific clinical, pathological, or molecular characteristic among all patients or in each age stratum. For continuous variables, values are presented as the median and interquartile range (25th-75th percentile).

^b^To compare categorical data and continuous data between age groups, the Spearman correlation test was performed using raw age values as a continuous variable.

^c^The Spearman correlation test was performed with CIMP status as a 9-level ordinal variable.

^d^The Spearman correlation test was performed with LINE-1 methylation level as a continuous variable.

To examine whether age at diagnosis and other clinicopathological factors differed by patient generation, we analyzed the associations of year of diagnosis with these variables. Patients who were diagnosed more recently had older age at diagnosis (*P* < 0.0001), lower disease stage (*P* = 0.0011), and higher LINE-1 methylation level (*P* < 0.0001), but no significant correlation was observed between year of diagnosis and exposure factors such as body mass index (BMI), physical activity, alcohol, and smoking (Supplementary Table S6).

### Multivariable-adjusted analyses of patient age and tumor vessels

Age at diagnosis also showed statistically significant correlations with sex, BMI, tumor location, CIMP status, and LINE-1 methylation level (at the alpha level of 0.005). To control for confounding, we conducted multivariable-adjusted logistic regression model with the inverse probability weighting (IPW) method that assessed the correlation of age (continuous or categorical variables) with the overall CD34^+^, micro CD34^+^, and CD34^+^LAMB1^+^ vessel densities (categorical variables) (Table 2; details of all variables in the models are in Supplementary Table S7; cross-tabulation of age and vessel density categories is in Supplementary Table S8). While assessing statistical trends using age as a continuous variable, we also showed odds ratio (OR) effect sizes comparing age categories. Compared with patients aged ≥70 years, the multivariable-adjusted ORs for high overall CD34^+^ vessel density were 0.85 (95% confidence interval [CI], 0.74–0.99) for those aged 55–69 years and 0.63 (95% CI, 0.48-0.81) for those aged <55 years (*P*_trend_ < 0.0001, calculated with the continuous age variable). Compared with patients aged ≥70 years, the multivariable-adjusted ORs for high CD34^+^LAMB1^+^ vessel density were 0.56 (95% CI, 0.47–0.65) for those aged 55–69 years and 0.28 (95% CI, 0.20–0.40) for those aged <55 years (*P*_trend_ < 0.0001). In contrast, age was not associated with the micro CD34^+^ vessel density (*P*_trend_ = 0.11). Furthermore, to eliminate confounding effect of MSI-high status, we conducted logistic regression analyses limited to non-MSI-high CRC and yielded similar results (Table 2). Compared with non-MSI-high tumor patients aged ≥70 years, the multivariable-adjusted ORs for high overall CD34^+^ vessel density were 0.97 (95% CI, 0.82–1.15) for those aged 55–69 years and 0.61 (95% CI, 0.46–0.81) for those aged <55 years (*P*_trend_ < 0.0001). Compared with non-MSI-high tumor patients aged ≥70 years, the multivariable-adjusted ORs for high CD34^+^LAMB1^+^ vessel density were 0.61 (95% CI, 0.51–0.72) for those aged 55–69 years and 0.25 (95% CI, 0.17–0.37) for those aged <55 years (*P*_trend_ < 0.0001).

**Table 2:** Logistic regression analysis to assess the associations of age at diagnosis (predictor) with vessel densities (binary outcome variables) in overall cases and non-MSI-high tumors.

Variables	Univariable	*P* value^d^	Multivariable-adjusted	*P* value^d^
	odds ratio (95% CI)^a^		odds ratio (95% CI)^a,b,c^	
Overall cases				
High overall CD34^+^ vessel density				
Age at diagnosis		<0.0001		<0.0001
<55	0.58 (0.45–0.75)		0.63 (0.48–0.81)	
55-69	0.80 (0.70–0.93)		0.85 (0.74–0.99)	
≥70	1 (referent)		1 (referent)	
High micro CD34^+^ vessel density				
Age at diagnosis		0.15		0.11
<55	1.16 (0.91–1.49)		1.16 (0.90–1.49)	
55-69	0.83 (0.72–0.96)		0.84 (0.72–0.97)	
≥70	1 (referent)		1 (referent)	
High CD34^+^LAMB1^+^ vessel density				
Age at diagnosis		<0.0001		<0.0001
<55	0.29 (0.20–0.41)		0.28 (0.20–0.40)	
55-69	0.56 (0.48–0.65)		0.56 (0.47–0.65)	
≥70	1 (referent)		1 (referent)	
**Non-MSI-high tumors**				
High overall CD34^+^ vessel density				
Age at diagnosis		<0.0001		<0.0001
<55	0.61 (0.47–0.80)		0.61 (0.46–0.81)	
55-69	0.92 (0.79–1.08)		0.97 (0.82–1.15)	
≥70	1 (referent)		1 (referent)	
High micro CD34^+^ vessel density				
Age at diagnosis		0.87		0.15
<55	1.20 (0.92–1.56)		1.07 (0.81–1.41)	
55–69	1.01 (0.86–1.18)		0.95 (0.81–1.12)	
≥70	1 (referent)		1 (referent)	
High CD34^+^LAMB1^+^ vessel density				
Age at diagnosis		<0.0001		<0.0001
<55	0.26 (0.18–0.38)		0.25 (0.17–0.37)	
55–69	0.61 (0.51–0.82)		0.61 (0.51–0.72)	
≥70	1 (referent)		1 (referent)	

*CI* confidence interval, *CIMP* CpG island methylator phenotype, *LINE-1* long interspersed nucleotide element-1, *MSI* microsatellite instability.

^a^Inverse probability weighting method was applied to reduce bias due to the data availability after cancer diagnosis.

^b^The multivariable logistic regression model initially included age at diagnosis, sex, body mass index at diagnosis, pack-year of smoking before diagnosis, family history of colorectal cancer, MSI status (not in the non-MSI-high tumor analysis), CIMP status, LINE-1 methylation level, *KRAS* mutation, *BRAF* mutation, and *PIK3CA* mutation.

^c^A backward elimination with a threshold *P* of 0.1 was used to select variables for each final model. The final models, including all variables that remained after the selection procedures, are shown in Supplementary Table S7.

^d^*P*_trend_ was calculated with raw age value (year) as a continuous variable in the logistic regression model.

Sensitivity analyses using logistic regression models without the IPW method also showed results similar to the IPW-adjusted analyses (Supplementary Table S9). Features of cases categorized by overall CD34^+^ vessel density (quartiles) and CD34^+^LAMB1^+^ vessel density (negative, low, high) are shown in Supplementary Tables S10–11.

## Discussion

We conducted this study to evaluate the relationship between age at diagnosis and tumor vascular features in the colorectal cancer (CRC) microenvironment. In addition to the predefined age categories, we considered the “age continuum” in our assessments. We found that both overall CD34^+^ vessel density and CD34^+^LAMB1^+^ vessel density were inversely correlated with younger age at diagnosis, suggesting that younger-onset CRC might exhibit fewer those vessels (i.e., severer hypovascularity) than later-onset CRC. We further conducted analyses excluding potential confounding effect of MSI-high tumors; the results using only non-MSI-high tumors showed similar results. Vascular features of early-onset CRC are worth further investigation.

A global rise of early-onset CRC incidence has attracted significant attention [5–11, 15, 32]. Early-onset CRC has been associated with distal colon/rectal location, signet ring cell histology, advanced stage, LINE-1 DNA hypomethylation, and the tumor microenvironment containing abundant FAP^+^ fibroblasts, low counts of infiltrating lymphocytes, regulatory T cells, M1-like macrophages, CD14^+^HLA-DR^+^ mature monocytes/macrophages, and the absence/paucity of dominant T cell clones [12–14, 33, 34]. When assessing tumor features, it is important to control for MSI-high (mismatch repair deficiency) status, as it is associated with microenvironmental features and commonly observed in early-onset CRCs in Lynch syndrome. Given the role of tumor vasculature in transporting immune cells as well as oxygen and nutrients to tumor and stromal cells, reduced vascularization may contribute to the development of the unique immune microenvironment observed in younger-onset CRC.

It is important to identify new biomarkers, including tumor features associated with early-onset CRC. Such features can provide pathogenic insights. Certain tumor features, including LINE-1 or global DNA hypomethylation [35–37], immunosuppression [12, 14], and colibactin-induced mutational signatures (SBS88 and ID18) [38, 39], have been associated with early-onset CRC. These tumor features can be further investigated using the prospective cohort incident-tumor biobank method (PCIBM). A prior PCIBM-based study could link long-term alcohol intake and folate insufficiency with the incidence of LINE-1 hypomethylated CRC (i.e., a feature of early-onset CRC), supporting those exposures as potential risk factors for early-onset CRC [40]. Other studies could link long-term inflammatory diets and nonuse of aspirin (vs. regular aspirin use) with the incidence of CRC having immunosuppressed microenvironmental features, which are associated with early-onset CRC [41, 42]. Another PCIBM-based study [43] could link long-term western-style diets with the incidence of CRC having abundant *pks*^*+*^ *Escherichia coli*, which can cause colibactin-induced mutational signatures [44]. Therefore, utilizing biomarkers of early-onset CRC can open a new way of investigating the role of long-term risk factor exposures in early-onset cancer etiologies [32].

In recent years, our understanding of tumor vasculature has advanced significantly. Single-cell RNA sequencing has revealed that endothelial cells are more heterogeneous than previously recognized, particularly within the tumor microenvironment [4]. These cells exhibit diverse molecular and morphological features depending on their functional state. Multispectral immunofluorescence allows for the simultaneous in-situ detection of multiple protein targets in a single tissue section. In this study, we used two tip cell markers (KDR and LAMB1), a stalk cell marker (CD36), a venous marker (ACKR1), and a high-endothelial venule marker (MADCAM1) to characterize tumor vessels [2, 3, 45, 46]. Among these markers, tip and stalk cell markers, indicative of proliferative vessels, were often co-expressed within the same vessels, and a similar tendency was observed for venous and high endothelial venule markers.

The vessel density is an indicator of angiogenesis in several types of cancer. While both LAMB1 and KDR represent tip cell markers, they differ in their temporal and cellular patterns of expression, with LAMB1 being expressed earlier in the angiogenic process than KDR [2]. LAMB1 has been reported to be expressed in neovascular vessels within tumors, reflecting enhanced angiogenic states. Our study showed a lower CD34^+^LAMB1^+^ vessel density in younger patients, suggesting that tumors of younger patients may exhibit a less active angiogenic phenotype compared to later-onset cases [28]. It has been reported that Lamb1 (the mouse homolog of human LAMB1) expression is increased in endothelial cells from aged mice compared to those from young mice, and this increase has been attributed to aging and ischemic conditions [47].

Age-related dysfunction of endothelial cells affects blood vessels of all sizes, from large arteries to small capillaries, and inhibits both angiogenesis and vascular maturation. However, the specific impact of aging on tumor vasculature remains poorly understood [48]. In the present study, the increase in overall vessel density and tip cells with increasing age suggests that age-related endothelial cell damage may primarily impair vessel maturation rather than neovascularization within the tumor microenvironment. Notably, prior studies showed that vessel density in the non-neoplastic gut did not significantly vary by age, indicating that the observed age-related vascular features may be specific to the tumor microenvironment [49].

How the tumor vessel phenotype is altered by host and/or tumor factors remains to be elucidated. While age-related changes in preexisting blood vessels and tissue can affect tumor vasculature as described above, angiogenic factors secreted by tumor, immune, and other stromal cells also influence tumor vessel formation [15, 50]. Since host tissues and cancer cells interact in complex manner to form the microenvironment, further investigation is needed to elucidate tumor vasculature phenotypes according to age.

Although available evidence is limited, studies suggest that pretreatment vessel density in tumor tissue may serve as a predictor of treatment response. Patients with higher vessel density may experience greater survival benefits from anti-VEGF inhibitors, whereas the efficacy of chemoradiotherapy may be lower in such cases [51, 52]. In light of these reports and our findings, it is possible that early-onset CRC patients with hypovascular tumor microenvironment may potentially derive relatively greater benefit from chemoradiotherapy than from anti-VEGF therapy, although further research is needed in this area.

Our study has limitations. First, the number of early-onset cases (age under 50) was not large. Therefore, to enhance statistical power, we carefully evaluated the age continuum model using age at diagnosis as a continuous variable and examined a combined group of “age under 50” and “age 50–54”. Second, the two U.S.-wide population-based prospective cohort studies with (young to middle-aged) adults started in 1976 and 1986. With this design, we did not have more recent birth cohorts, such as those born after 1970. Nonetheless, our previous studies using the same cohorts have revealed tumoral features of early-onset CRCs such as tumor LINE-1 hypomethylation [36] and immunosuppressed microenvironmental features [12]. These features have been replicated in independent studies [14, 35]. These studies have attested that our large-scale population-based prospective cohort studies might enable us to find new features of early-onset CRC. Third, as our study was based on the prospective cohort studies of predominantly White health professional populations, the generalizability of our findings needs to be tested. Furthermore, most patients under 55 years of age were diagnosed before 2000. Therefore, further validation studies should be performed in more recent patient populations. Fourth, the tissue microarrays were used, which might have resulted in a lower detection rate for certain types of vessels. Specifically, because high-endothelial venules are predominantly localized in tertiary lymphoid structures, MADCAM1^+^ vessel density would be lower than measured at the peritumoral regions [53]. However, our tissue microarray blocks were constructed using multiple tissue cores from tumor-invading edges as well as tumor centers of each tumor. Furthermore, a previous study reported that high endothelial venous density measured in both the intratumoral and peritumoral regions did not correlate with age at diagnosis, which is consistent with our results [54]. Fifth, since the machine-learning-based morphological classification relied on pathologist supervision, some degree of subjectivity in the morphological categories cannot be excluded. However, the high reproducibility between the two pathologists (91% concordance rate; unweighted Cohen’s kappa = 0.88) suggests that the classification remains reasonably acceptable and reliable.

The current study has strengths. First, we employed objective, automated computational image analysis combined with supervised machine learning to quantify vessel density, minimizing inter-observer variability commonly associated with manual vessel counting [55, 56]. This methodology has enhanced the accuracy and reproducibility of our findings and represents a significant advancement over prior studies that relied on manual or semi-quantitative assessments [57–59]. To our knowledge, this is the first report to examine multiple endothelial cell markers in situ across the large CRC cohorts. Second, we used the PCIBM [18–20] and collected tumor specimens from hundreds of hospitals across the United States, which allowed us to minimize case selection bias associated with a limited number of hospitals. Third, the PCIBM also allowed us to leverage the database of the entire cohort studies and adjust for selection bias due to tissue data availability using the IPW method. Fourth, the PCIBM further enabled us to conduct multivariate analysis incorporating a wide range of clinical and molecular pathological data to control for confounding. Because both systemic vasculature and tumor-associated vessels may be affected by various factors, our adjusted models provided a more rigorous assessment of the relationship between age and tumor vascular features.

In conclusion, this study suggests that hypovascularities of both overall CD34^+^ and CD34^+^LAMB1^+^ vessels of the CRC microenvironment may be histological features of younger-onset or early-onset CRC, if validated by independent studies. Differences in tumor vasculature may contribute to the formation of tumor microenvironment of early-onset CRC. It is important to identify new biomarkers (such as tumor characteristics) of early-onset CRC, because those can be utilized to decipher the etiologies of early-onset CRC [32].

## Supplementary information

Supplementary material

## Data Availability

The data underlying this article are not publicly available and may be obtained upon a reasonable request.

## Code availability

Analyses in this study were performed using R software version 4.4.0, MATLAB software R2023b, and SAS version 9.4. The custom scripts used for data processing and statistical analyses are not publicly available due to ongoing related projects, but they are available from the corresponding author upon reasonable request.
