# Supplementary material for: Tumor vessel phenotype in colorectal cancer microenvironment according to age at diagnosis
Source: Br J Cancer. 2026 Mar 25;134(10):1375–86. doi: 10.1038/s41416-026-03373-6 (PMC13133279; doi:10.1038/s41416-026-03373-6)
Supplement: Supplementary file 1 — Supplementary material [file 41416_2026_3373_MOESM1_ESM.docx]

**Supplementary contents**

Tumor vessel phenotype in colorectal cancer microenvironment according to age at diagnosis

**Authors**

Kosuke Matsuda^1^, Satoko Ugai^1,2^, Satoshi Miyahara^1^, Qian Yao^1^, Jules Cazaubiel^3^,

Nobuhiro Nakazawa^1^, Mayu Higashioka^1^, Yuxue Zhong^1^, Andrew T. Chan^2,4,5^,

Jeffrey A. Meyerhardt^3^, Kimmie Ng^3^, Mingyang Song^2,4,6^, Juha P. Väyrynen^7^,

Jonathan A. Nowak^3^, Marios Giannakis^3,8^, Tomotaka Ugai^1,2,9^, Shuji Ogino^1,2,8,10,11^

**Author affiliations**

1. Program in MPE Molecular Pathological Epidemiology, Department of Pathology, Brigham and Women’s Hospital, and Harvard Medical School, Boston, MA, USA
2. Department of Epidemiology, Harvard T.H. Chan School of Public Health, Boston, MA, USA
3. Department of Medical Oncology, Dana-Farber Cancer Institute, Boston, Massachusetts, USA
4. Clinical and Translational Epidemiology Unit, Massachusetts General Hospital and Harvard Medical School, Boston, Massachusetts, USA
5. Division of Gastroenterology, Massachusetts General Hospital and Harvard Medical School, Boston, Massachusetts, USA
6. Department of Nutrition, Harvard T.H. Chan School of Public Health, Boston, MA, USA
7. Translational Medicine Research Unit, University of Oulu, Medical Research Center Oulu, and Oulu University Hospital, Oulu, Finland
8. Broad Institute of MIT and Harvard, Cambridge, MA, USA
9. Division of Integrative Cancer Research, National Cancer Center Research Institute, Tokyo, Japan.
10. Department of Medicine, Brigham and Women’s Hospital and Harvard Medical School, Boston, MA, USA
11. Institute of Science Tokyo, Tokyo, Japan

**Supplementary Table S1.** List of antibodies and fluorophores used in the multispectral immunofluorescence.

| Order | Marker | Clone | Supplier, catalogue number [lot number] | Antibody dilution | Fluorophore  [lot number]  (dilution) | Fluorophore incubation time |
| --- | --- | --- | --- | --- | --- | --- |
| 1 | ACKR1 (DARC) | Polyclonal | Millipore Sigma, HPA017672 [000008347] | 1:100 | Opal 570  [20232104]  (1:100) | 15 min |
| 2 | KDR (VEGFR2) | 55B11 | Cell Signaling Technology, #2479 [21] | 1:125 | Opal 480  [20232307]  (1:100) | 30 min |
| 3 | MADCAM1 | Polyclonal | Millipore Sigma, HPA077998 [R113657] | 1:100 | Opal 520  [20234625]  (1:100) | 10 min |
| 4 | CD36 | Polyclonal | Millipore Sigma, HPA002018 [000001270] | 1:100 | Opal 620  [20232306]  (1:100) | 15 min |
| 5 | LAMB1 | D-9 | Santa Cruz Biotechnology,  sc-17763 [C1605] | 1:25 | Opal 650  [20234830]  (1:100) | 30 min |
| 6 | CD34 | QBEnd10 | DAKO, M7165 [41640452] | 1:100 | Opal 690  [20232205]  (1:100) | 30 min |
| 7 | KRT | AE1/AE3; C11 | DAKO, M3515 [11683567]; Cell Signaling Technology, #4545 [17] | 1:40; 1:400 | Opal 780  [20234803]  (1:25) | 15 min |

**Supplementary Table S2.** Variable importance of vascular morphological features as determined by the random forest model.

| Feature | Mean decrease  in accuracy | Mean decrease  in Gini impurity | Class-specific mean decrease in accuracy | | | |
| --- | --- | --- | --- | --- | --- | --- |
|  |  |  | Micro | Collapsed | Patent | Irregular |
| Area | 21.5 | 16.1 | 11.9 | 10.0 | 17.8 | 12.7 |
| Circularity | 31.7 | 21.1 | 12.2 | 21.7 | 10.8 | 25.2 |
| ConvexArea | 24.5 | 25.7 | 16.2 | 11.9 | 14.9 | 18.9 |
| Eccentricity | 36.7 | 21.2 | 14.9 | 32.3 | 12.2 | 13.4 |
| EquivDiameter | 21.1 | 17.0 | 12.5 | 11.5 | 16.5 | 13.3 |
| Extent | 25.2 | 17.4 | 10.8 | 12.0 | 12.9 | 18.8 |
| MajorAxisLength | 33.7 | 46.6 | 30.9 | 24.7 | 21.3 | 25.6 |
| MinorAxisLength | 69.8 | 60.0 | 11.1 | 59.6 | 36.8 | 46.7 |
| Perimeter | 25.5 | 35.9 | 19.5 | 13.3 | 18.2 | 23.5 |
| Solidity | 57.2 | 38.2 | 4.5 | 10.2 | 21.3 | 59.8 |

**Supplementary Table S3.** Confusion matrix of the random forest model on the training and validation sets for morphological classification of CD34^+^ vessels.

| Ground truth | | Predicted class | | | |
| --- | --- | --- | --- | --- | --- |
|  |  | Micro | Collapsed | Patent | Irregular |
| Training set | |  |  |  |  |
|  | Micro | 99 | 0 | 0 | 1 |
|  | Collapsed | 0 | 97 | 0 | 3 |
|  | Patent | 0 | 3 | 91 | 6 |
|  | Irregular | 0 | 3 | 2 | 95 |
|  |  |  |  |  |  |
| Validation set | |  |  |  |  |
|  | Micro | 97 | 1 | 1 | 1 |
|  | Collapsed | 1 | 90 | 0 | 9 |
|  | Patent | 0 | 2 | 85 | 13 |
|  | Irregular | 0 | 2 | 4 | 94 |

**Supplementary Table S4.** Clinical, pathological, and molecular characteristics of colorectal cancer cases according to age at diagnosis.

|  |  |  | |  | | | | Age at diagnosis | | | |  | |
| --- | --- | --- | --- | --- | --- | --- | --- | --- | --- | --- | --- | --- | --- |
| Characteristics ^a^ | | All cases | <50 | | | 50-54 | | | | 55-69 | | | ≥70 |
|  |  | N=843 | N=14 | | | N=38 | | | | N=400 | | | N=391 |
| Sex | |  |  | | |  | | | |  | | |  |
|  | Female (NHS) | 468 (56%) | 13 (93%) | | | 27 (71%) | | | | 249 (62%) | | | 179 (46%) |
|  | Male (HPFS) | 375 (44%) | 1 (7%) | | | 11 (29%) | | | | 151 (38%) | | | 212 (54%) |
| BMI at diagnosis (kg/m^2^) | | 26 (24-29) | 25 (23-30) | | | 26 (24-29) | | | | 26 (24-29) | | | 25 (23-28) |
| Prediagnostic physical activity (METS hour/week) | | 13 (3.7-29) | 12 (5.1-29) | | | 11 (6.0-17) | | | | 12 (3.6-31) | | | 13 (3.8-29) |
| Prediagnostic alcohol consumption (g/day) | | 2.3 (0-12) | 4.1 (0-8.8) | | | 1.8 (0-6.8) | | | | 2.4 (0-11) | | | 2.2 (0-13) |
| Prediagnostic pack-year of smoking | | 7.0 (0-30) | 2.0 (0-14) | | | 0 (0-12) | | | | 7.5 (0-31) | | | 10 (0-33) |
| Family history of colorectal cancer in a first-degree relative | | | | | | | | |  | | | | |
|  | Absent | 659 (79%) | 10 (71%) | | | 29 (76%) | | | | 315 (79%) | | | 305 (78%) |
|  | Present | 176 (21%) | 4 (29%) | | | 8 (21%) | | | | 82 (21%) | | | 82 (21%) |
| Tumor location | |  |  | | |  | | | |  | | |  |
|  | Proximal colon | 420 (50%) | 4 (29%) | | | 15 (39%) | | | | 184 (46%) | | | 217 (55%) |
|  | Distal colon | 248 (29%) | 7 (50%) | | | 14 (37%) | | | | 132 (33%) | | | 95 (24%) |
|  | Rectum | 172 (20%) | 3 (21%) | | | 9 (24%) | | | | 83 (21%) | | | 77 (20%) |
| AJCC disease stage | |  |  | | |  | | | |  | | |  |
|  | I | 183 (22%) | 5 (36%) | | | 6 (16%) | | | | 74 (19%) | | | 98 (25%) |
|  | II | 254 (30%) | 3 (21%) | | | 7 (18%) | | | | 123 (31%) | | | 121 (31%) |
|  | III | 226 (27%) | 4 (29%) | | | 18 (47%) | | | | 110 (28%) | | | 94 (24%) |
|  | IV | 119 (14%) | 2 (14%) | | | 5 (13%) | | | | 67 (17%) | | | 45 (12%) |
| Tumor differentiation | |  |  | | |  | | | |  | | |  |
|  | Well/ Moderate | 773 (92%) | 13 (93%) | | | 35 (92%) | | | | 369 (92%) | | | 356 (91%) |
|  | Poor | 69 (8%) | 1 (7%) | | | 3 (8%) | | | | 31 (8%) | | | 34 (9%) |
| MSI status | |  |  | | |  | | | |  | | |  |
|  | Non-MSI-high | 674 (80%) | 14 (100%) | | | 32 (84%) | | | | 328 (82%) | | | 300 (77%) |
|  | MSI-high | 143 (17%) | 0 | | | 5 (13%) | | | | 62 (16%) | | | 76 (19%) |
| CIMP status | |  |  | | |  | | | |  | | |  |
|  | Negative/ Low | 633 (81%) | 13 (93%) | | | 34 (89%) | | | | 322 (85%) | | | 264 (76%) |
|  | High | 146 (19%) | 1 (7%) | | | 4 (11%) | | | | 57 (15%) | | | 84 (24%) |
| LINE-1 methylation level | | | | |  | |  | | | |  | | |
|  | ≤55 | 180 (21%) | 4 (29%) | | | 9 (24%) | | | | 88 (22%) | | | 79 (20%) |
|  | 55-65 | 329 (39%) | 6 (43%) | | | 15 (39%) | | | | 167 (42%) | | | 141 (36%) |
|  | >65 | 325 (39%) | 4 (29%) | | | 13 (34%) | | | | 142 (36%) | | | 166 (42%) |
| *KRAS* mutation | |  |  | | |  | | | |  | | |  |
|  | Wild type | 490 (58%) | 10 (71%) | | | 27 (71%) | | | | 229 (57%) | | | 224 (57%) |
|  | Mutated | 328 (39%) | 4 (29%) | | | 9 (24%) | | | | 160 (40%) | | | 155 (40%) |
| *BRAF* mutation | |  |  | | |  | | | |  | | |  |
|  | Wild type | 692 (82%) | 12 (86%) | | | 31 (82%) | | | | 330 (83%) | | | 319 (82%) |
|  | Mutated | 132 (16%) | 2 (14%) | | | 6 (16%) | | | | 62 (16%) | | | 62 (16%) |
| *PIK3CA* mutation | |  |  | | |  | | | |  | | |  |
|  | Wild type | 641 (76%) | 13 (93%) | | | 32 (84%) | | | | 292 (73%) | | | 304 (78%) |
|  | Mutated | 127 (15%) | 1 (7%) | | | 3 (8%) | | | | 61 (15%) | | | 62 (16%) |
| Tumor-infiltrating lymphocytes | | | | |  | |  | | | |  | | |
|  | Absent/ Low | 606 (72%) | 13 (93%) | | | 29 (76%) | | | | 291 (73%) | | | 273 (70%) |
|  | Intermediate | 132 (16%) | 1 (7%) | | | 5 (13%) | | | | 58 (15%) | | | 68 (17%) |
|  | High | 97 (12%) | 0 | | | 4 (11%) | | | | 44 (11%) | | | 49 (13%) |
|  | | | | |  | |  | | | |  | | |
|  | | | | |  | |  | | | |  | | |
| Intratumoral periglandular reaction | | | | |  | |  | | | |  | | |
|  | Absent/ Low | 110 (13%) | 2 (14%) | | | 3 (8%) | | | | 43 (11%) | | | 62 (16%) |
|  | Intermediate | 622 (74%) | 12 (86%) | | | 32 (84%) | | | | 307 (77%) | | | 271 (69%) |
|  | High | 104 (12%) | 0 | | | 3 (8%) | | | | 271 (68%) | | | 57 (15%) |
| Peritumoral lymphocytic reaction | | | | |  | |  | | | |  | | |
|  | Absent/ Low | 128 (15%) | 2 (14%) | | | 3 (8%) | | | | 44 (11%) | | | 79 (20%) |
|  | Intermediate | 574 (68%) | 11 (79%) | | | 31 (82%) | | | | 303 (76%) | | | 229 (59%) |
|  | High | 131 (16%) | 1 (7%) | | | 4 (11%) | | | | 45 (11%) | | | 81 (21%) |
| Crohn's-like lymphoid reaction | | | | |  | |  | | | |  | | |
|  | Absent/ Low | 522 (62%) | 10 (71%) | | | 19 (50%) | | | | 249 (62%) | | | 244 (62%) |
|  | Intermediate | 126 (15%) | 1 (7%) | | | 2 (5%) | | | | 60 (15%) | | | 63 (16%) |
|  | High | 56 (7%) | 0 | | | 3 (8%) | | | | 23 (6%) | | | 30 (8%) |
| Vessel density (/mm^2^) | | | | |  | |  | | | |  | | |
|  | Overall CD34^+^ | 197 (133-262) | 182 (148-226) | | | 179 (130-241) | | | | 191 (127-254) | | | 205 (140-277) |
|  | Collapsed CD34^+^ | 34 (21-51) | 29 (22-41) | | | 34 (21-55) | | | | 33 (21-48) | | | 36 (22-54) |
|  | Micro CD34^+^ | 93 (56-134) | 96 (62-116) | | | 87 (48-125) | | | | 92 (52-126) | | | 95 (59-139) |
|  | Patent CD34^+^ | 27 (15-39) | 24 (12-36) | | | 27 (16-35) | | | | 25 (14-39) | | | 27 (17-39) |
|  | CD34^+^ACKR1^+^ | 0 (0-5.2) | 0.49 (0-8.7) | | | 0 (0-5.5) | | | | 0 (0-4.9) | | | 0 (0-5.4) |
|  | CD34^+^CD36^+^ | 1.7 (0-8.5) | 0 (0-2.8) | | | 1.1 (0-4.4) | | | | 1.6 (0-7.5) | | | 1.7 (0-9.5) |
|  | CD34^+^KDR^+^ | 5.0 (0-22) | 4.7 (0-34) | | | 4.7 (0-18) | | | | 5.0 (0-21) | | | 5.0 (0-23) |
|  | CD34^+^LAMB1^+^ | 2.4 (0-12) | 2.7 (0-4.5) | | | 0 (0-3.6) | | | | 2.1 (0-9.0) | | | 3.5 (0-16) |
|  | CD34^+^MADCAM1^+^ | 0 (0-2.3) | 0 (0-6.1) | | | 0 (0-0) | | | | 0 (0-2.6) | | | 0 (0-2.3) |

^a^ Percentage indicates the proportion of patients with a specific clinical, pathological, or molecular characteristic among all patients or in the strata of age at diagnosis. For continuous variables, values are presented as the median and interquartile range (25th-75th percentile).

Abbreviations: AJCC, American Joint Committee on Cancer; BMI, body mass index; CIMP, CpG island methylator phenotype; HPFS, Health Professionals Follow-up Study; LINE-1, long interspersed nucleotide element-1; MET, metabolic equivalent; MET, metabolic equivalent of task; MSI, microsatellite instability; NHS, Nurses’ Health Study.**Supplementary Table S5.** Clinical, pathological, and molecular characteristics of colorectal cancer cases according to age at diagnosis in non-MSI-high tumors

|  |  | |  | Age at diagnosis | | | | | |  |
| --- | --- | --- | --- | --- | --- | --- | --- | --- | --- | --- |
| Characteristics ^a^ | | | All cases | | <55 | | 55-69 | | ≥70 | *P* value ^b^ |
|  |  | | N=674 | | N=46 | | N=328 | | N=300 |  |
|  | | |  | |  | |  | |  |  |
| Sex | | |  | |  | |  | |  | <0.0001 |
|  | Female (NHS) | | 354 (52%) | | 36 (78%) | | 203 (62%) | | 115 (38%) |  |
|  | Male (HPFS) | | 320 (48%) | | 10 (22%) | | 125 (38%) | | 185 (62%) |  |
| BMI preceding diagnosis (kg/m^2^) | | | 26 (24-29) | | 26 (24-29) | | 26 (24-29) | | 25 (23-28) | 0.018 |
| Prediagnostic physical activity (METS hours/week) | | | 13 (3.7-30) | | 11 (6.0-18) | | 12 (3.5-30) | | 14 (3.9-35) | 0.85 |
| Prediagnostic alcohol consumption (g/day) | | | 2.9 (0-13) | | 2.6 (0-8.1) | | 2.6 (0-12) | | 3.4 (0-14) | 0.35 |
| Prediagnostic pack-year of smoking | | | 7.0 (0-28) | | 0 (0-13) | | 8.0 (0-31) | | 7.0 (0-28) | 0.37 |
| Family history of colorectal  cancer in a first-degree relative | | | | | | | | | | 0.82 |
|  | Absent | | 529 (80%) | | 34 (76%) | | 255 (78%) | | 240 (81%) |  |
|  | Present | | 136 (20%) | | 11 (24%) | | 68 (22%) | | 57 (19%) |  |
| Tumor location | | |  | |  | |  | |  | 0.039 |
|  | Proximal colon | | 281 (42%) | | 14 (30%) | | 128 (39%) | | 139 (47%) |  |
|  | Distal colon | | 228 (34%) | | 20 (44%) | | 119 (37%) | | 89 (30%) |  |
|  | Rectum | | 160 (24%) | | 12 (26%) | | 78 (24%) | | 70 (23%) |  |
| AJCC disease stage | | |  | |  | |  | |  | 0.0036 |
|  | I | | 148 (24%) | | 10 (23%) | | 60 (20%) | | 78 (28%) |  |
|  | II | | 172 (28%) | | 8 (18%) | | 91 (30%) | | 73 (27%) |  |
|  | III | | 195 (31%) | | 19 (43%) | | 94 (30%) | | 82 (30%) |  |
|  | IV | | 109 (17%) | | 7 (16%) | | 61 (20%) | | 41 (15%) |  |
| Tumor differentiation | | |  | |  | |  | |  | 0.62 |
|  | Well/ Moderate | | 671 (96%) | | 43 (93%) | | 316 (97%) | | 285 (95%) |  |
|  | Poor | | 27 (4%) | | 3 (7%) | | 10 (3%) | | 14 (5%) |  |
| CIMP status | | |  | |  | |  | |  | 0.12 ^c^ |
|  | Negative/ Low | | 583 (93%) | | 43 (93%) | | 296 (95%) | | 244 (91%) |  |
|  | High | | 44 (7%) | | 3 (7%) | | 16 (5%) | | 25 (9%) |  |
| LINE-1 methylation level | | | | | |  | |  | | 0.060 ^d^ |
|  | ≤55% | | 154 (23%) | | 13 (28%) | | 71 (21%) | | 70 (23%) |  |
|  | 55-65% | | 287 (43%) | | 20 (44%) | | 146 (43%) | | 121 (40%) |  |
|  | >65% | | 231 (34%) | | 13 (28%) | | 121 (36%) | | 109 (36%) |  |
| *KRAS* mutation | | |  | |  | |  | |  | 0.034 |
|  | Wild type | | 368 (55%) | | 34 (76%) | | 180 (56%) | | 154 (52%) |  |
|  | Mutated | | 298 (45%) | | 11 (24%) | | 142 (44%) | | 145 (48%) |  |
| *BRAF* mutation | | |  | |  | |  | |  | 0.074 |
|  | Wild type | | 610 (92%) | | 39 (87%) | | 290 (90%) | | 281 (94%) |  |
|  | Mutated | | 56 (8%) | | 6 (13%) | | 33 (10%) | | 17 (6%) |  |
| *PIK3CA* mutation | | |  | |  | |  | |  | 0.17 |
|  | Wild type | | 524 (83%) | | 41 (93%) | | 246 (83%) | | 237 (83%) |  |
|  | Mutated | | 105 (17%) | | 3 (7%) | | 52 (17%) | | 50 (17%) |  |
| Tumor-infiltrating lymphocytes | | | | | |  | |  | | 0.43 |
|  | Absent/ Low | | 544 (82%) | | 40 (87%) | | 260 (81%) | | 244 (82%) |  |
|  | Intermediate | | 89 (13%) | | 5 (11%) | | 44 (14%) | | 40 (13%) |  |
|  | High | | 32 (5%) | | 1 (2%) | | 16 (5%) | | 15 (5%) |  |
|  | | | | | |  | |  | |  |
|  | | | | | |  | |  | |  |
|  | | | | | |  | |  | |  |
| Intratumoral periglandular reaction | | | | | |  | |  | | 0.72 |
|  | Absent/ Low | | 98 (15%) | | 5 (11%) | | 39 (12%) | | 54 (18%) |  |
|  | Intermediate | | 515 (77%) | | 40 (87%) | | 260 (81%) | | 215 (72%) |  |
|  | High | | 53 (8%) | | 1 (2%) | | 22 (7%) | | 30 (10%) |  |
| Peritumoral lymphocytic reaction | | | | | |  | |  | | 0.30 |
|  | Absent/ Low | | 110 (17%) | | 5 (11%) | | 38 (12%) | | 79 (20%) |  |
|  | Intermediate | | 480 (72%) | | 38 (83%) | | 256 (80%) | | 229 (59%) |  |
|  | High | | 73 (11%) | | 3 (6%) | | 25 (8%) | | 81 (21%) |  |
| Crohn's-like lymphoid reaction | | | | | |  | |  | | 0.95 |
|  | Absent/ Low | | 457 (81%) | | 28 (85%) | | 220 (80%) | | 209 (81%) |  |
|  | Intermediate | | 86 (15%) | | 3 (9%) | | 44 (16%) | | 39 (15%) |  |
|  | High | | 22 (4%) | | 2 (6%) | | 11 (4%) | | 9 (4%) |  |
| Vessel density (/mm^2^) | |  | | | |  | |  | |  |
|  | Overall CD34^+^ | | 192 (131-256) | | 182 (138-241) | | 189 (127-251) | | 199 (133-268) | 0.043 |
|  | Collapsed CD34^+^ | | 31 (19-47) | | 26 (15-36) | | 34 (21-47) | | 34 (22-52) | 0.079 |
|  | Micro CD34^+^ | | 89 (55-130) | | 89 (47-120) | | 89 (52-126) | | 88 (56-133) | 0.093 |
|  | Patent CD34^+^ | | 26 (15-39) | | 26 (15-36) | | 25 (14-38) | | 27 (16-39) | 0.26 |
|  | CD34^+^ACKR1^+^ | | 0 (0-5.5) | | 0 (0-5.6) | | 0 (0-5.5) | | 0 (0-5.4) | 0.97 |
|  | CD34^+^CD36^+^ | | 1.7 (0-8.6) | | 0 (0-4.4) | | 1.7 (0-7.7) | | 1.9 (0-9.0) | 0.43 |
|  | CD34^+^KDR^+^ | | 4.7 (0-20) | | 4.7 (0-24) | | 4.8 (0-20) | | 4.5 (0-20) | 0.72 |
|  | CD34^+^LAMB1^+^ | | 2.4 (0-11) | | 0.6 (0-4.4) | | 2.2 (0-8.8) | | 3.5 (0-16) | 0.012 |
|  | CD34^+^MADCAM1^+^ | | 0 (0-3.7) | | 0 (0-2.5) | | 0 (0-4.1) | | 0 (0-5.4) | 0.33 |

^a^ Percentage indicates the proportion of patients with a specific clinical, pathological, or molecular characteristic among all patients or in each age stratum. For continuous variables, values are presented as the median and interquartile range (25th-75th percentile).

^b^ To compare categorical data and continuous data between age groups, the Spearman correlation test was performed using raw age values as a continuous variable.

^c^ The Spearman correlation test was performed with CIMP status as a 9-level ordinal variable.

^d^ The Spearman correlation test was performed with LINE-1 methylation level as a continuous variable.

Abbreviations: AJCC, American Joint Committee on Cancer; BMI, body mass index; CIMP, CpG island methylator phenotype; HPFS, Health Professionals Follow-up Study; LINE-1, long interspersed nucleotide element-1; METS, metabolic equivalent of task score; MSI, microsatellite instability; NHS, Nurses’ Health Study.

**Supplementary Table S6**. Clinical, pathological, and molecular characteristics of colorectal cancer cases according to year of colorectal cancer diagnosis.

|  | | Year of diagnosis | | | |  |
| --- | --- | --- | --- | --- | --- | --- |
| Characteristics ^a^ | | All cases | 1980-1989 | 1990-1999 | 2000-2014 | *P* value ^b^ |
|  |  | N=843 | N=83 | N=406 | N=354 |  |
| Age at diagnosis (years old) | | |  |  |  | <0.0001 |
|  | <50 | 14 (2%) | 7 (8%) | 7 (2%) | 0 |  |
|  | 50-54 | 38 (5%) | 18 (22%) | 19 (5%) | 1 (0.3%) |  |
|  | 55-69 | 400 (47%) | 51 (62%) | 228 (56%) | 121 (34%) |  |
|  | ≥70 | 391 (46%) | 7 (8%) | 152 (37%) | 232 (66%) |  |
| Sex | |  |  |  |  | 0.13 |
|  | Female | 468 (56%) | 30 (36%) | 177 (44%) | 170 (48%) |  |
|  | Male | 375 (44%) | 53 (64%) | 229 (56%) | 184 (52%) |  |
| Family history of colorectal  cancer in a first-degree relative | | | | |  | 0.52 |
|  | Absent | 659 (79%) | 64 (81%) | 314 (78%) | 281 (80%) |  |
|  | Present | 176 (21%) | 15 (19%) | 89 (22%) | 72 (20%) |  |
| Body mass index preceding diagnosis (kg/m^2^) | | 26 (24-29) | 26 (24-28) | 26 (24-28) | 26 (24-30) | 0.13 |
| Prediagnostic physical activity (METS hour/week) | | 13 (3.7-29) | 8.8 (3.7-24) | 13 (3.5-29) | 13 (3.9-30) | 0.74 |
| Prediagnostic pack-year of smoking | | 7.0 (0-30) | 4.0 (0-29) | 8.0 (0-33) | 7.0 (0-27) | 0.89 |
| Prediagnostic alcohol (g/day) | | 2.3 (0-12) | 2.4 (0-14) | 2.0 (0-12) | 2.3 (0-11) | 0.35 |
| Tumor location | |  |  |  |  | 0.011 |
|  | Proximal colon | 420 (50%) | 32 (39%) | 200 (48%) | 188 (54%) |  |
|  | Distal colon | 248 (30%) | 34 (41%) | 132 (32%) | 92 (26%) |  |
|  | Rectum | 172 (20%) | 17 (20%) | 84 (20%) | 71 (20%) |  |
| AJCC disease stage | |  |  |  |  | 0.0011 |
|  | I | 183 (23%) | 18 (23%) | 82 (22%) | 83 (26%) |  |
|  | II | 254 (32%) | 22 (28%) | 123 (32%) | 109 (34%) |  |
|  | III | 226 (29%) | 20 (26%) | 111 (29%) | 95 (29%) |  |
|  | IV | 119 (15%) | 18 (23%) | 64 (17%) | 37 (11%) |  |
| Tumor grade | |  |  |  |  | 0.36 |
|  | Well/ Moderate | 773 (92%) | 75 (90%) | 369 (91%) | 329 (93%) |  |
|  | Poor | 69 (8%) | 8 (10%) | 36 (9%) | 25 (7%) |  |
| MSI status | |  |  |  |  | 0.021 |
|  | Non-MSI-high | 674 (82%) | 70 (86%) | 337 (84%) | 267 (80%) |  |
|  | MSI-high | 143 (18%) | 11 (14%) | 66 (16%) | 66 (20%) |  |
| CIMP status | |  |  |  |  | 0.019 ^c^ |
|  | Negative/ Low | 633 (81%) | 75 (91%) | 330 (83%) | 228 (76%) |  |
|  | High | 146 (19%) | 7 (19%) | 67 (17%) | 72 (24%) |  |
| LINE-1 methylation level | | |  |  |  | <0.0001 ^d^ |
|  | ≤55% | 182 (22%) | 20 (24%) | 109 (27%) | 53 (15%) |  |
|  | 55-65% | 331 (39%) | 40 (48%) | 166 (42%) | 125 (35%) |  |
|  | >65% | 328 (39%) | 23 (28%) | 125 (31%) | 176 (50%) |  |
| *KRAS* mutation | |  |  |  |  | 0.43 |
|  | Wild type | 490 (60%) | 51 (63%) | 233 (58%) | 206 (61%) |  |
|  | Mutated | 328 (40%) | 30 (37%) | 166 (42%) | 132 (39%) |  |
| *BRAF* mutation | |  |  |  |  | 0.45 |
|  | Wild type | 692 (84%) | 69 (87%) | 338 (84%) | 285 (83%) |  |
|  | Mutated | 132 (16%) | 11 (13%) | 63 (16%) | 58 (17%) |  |
| *PIK3CA* mutation | |  |  |  |  | 0.63 |
|  | Wild type | 641 (83%) | 56 (80%) | 307 (86%) | 278 (82%) |  |
|  | Mutated | 127 (17%) | 14 (20%) | 52 (14%) | 61 (18%) |  |

^a^ Percentage indicates the proportion of patients with a specific clinical, pathological, or molecular characteristic among all patients or in each age stratum. For continuous variables, values are presented as the median and interquartile range (25th-75th percentile).

^b^ To compare categorical data and continuous data between birth year groups, the Spearman correlation test was performed using raw year of diagnosis values as a continuous variable.

^c^ The Spearman correlation test was performed with CIMP status as a 9-level ordinal variable.

^d^ The Spearman correlation test was performed with LINE-1 methylation level as a continuous variable.

Abbreviations: AJCC, American Joint Committee on Cancer; CIMP, CpG island methylator phenotype; HPFS, Health Professionals Follow-up Study; LINE-1, long interspersed nucleotide element-1; METS, metabolic equivalent of task score; MSI, microsatellite instability; NHS, Nurses’ Health Study.

**Supplementary Table S7.** Details of the multivariable-adjusted logistic regression models to assess the association of age at diagnosis (predictor) with vessel densities (binary outcome variables) in overall cases.

| Variables in the final model | | | Univariable-unadjusted  odds ratio (95% CI) ^a^ | *P* value | Multivariable-adjusted  odds ratio (95% CI) ^a,b,c^ | | *P* value | |
| --- | --- | --- | --- | --- | --- | --- | --- | --- |
| High overall CD34^+^ vessel density | | | |  |  | |  | |
|  | Age at diagnosis | |  | <0.0001 ^d^ |  | | <0.0001 ^d^ | |
|  |  | <55 | 0.58 (0.45 - 0.75) |  | 0.63 (0.48 - 0.81) | |  | |
|  |  | 55-69 | 0.80 (0.70 - 0.93) |  | 0.85 (0.74 - 0.99) | |  | |
|  |  | ≥70 | Referent |  | Referent | |  | |
|  | Sex | |  | <0.0001 |  | | 0.0065 ^d^ | |
|  |  | Female | 0.72 (0.62 - 0.83) |  | 0.75 (0.65 - 0.87) | |  | |
|  |  | Male | Referent |  | Referent | |  | |
|  | MSI status | |  | <0.0001 |  | | <0.0001 ^d^ | |
|  |  | Non-MSI-high | Referent |  | Referent | |  | |
|  |  | MSI-high | 1.46 (1.21 - 1.76) |  | 1.92 (1.53 - 2.40) | |  | |
|  | *KRAS* mutation | |  | 0.0002 |  | | <0.0001 ^d^ | |
|  |  | Wild type | Referent |  | Referent | |  | |
|  |  | Mutated | 0.77 (0.67 - 0.88) |  | 0.65 (0.56 - 0.75) | |  | |
|  | *BRAF* mutation | |  | 0.0014 |  | | <0.0001 ^d^ | |
|  |  | Wild type | Referent |  | Referent | |  | |
|  |  | Mutated | 0.73 (0.61 - 0.89) |  | 0.47 (0.37 - 0.60) | |  | |
|  |  |  |  |  |  | |  | |
| High micro CD34^+^ vessel density | | | |  |  | |  | |
|  | Age at diagnosis | |  | 0.15 ^d^ |  | | 0.11 ^d^ | |
|  |  | <55 | 1.16 (0.91 - 1.49) |  | 1.16 (0.90 - 1.49) | |  | |
|  |  | 55-69 | 0.83 (0.72 - 0.96) |  | 0.84 (0.72 - 0.97) | |  | |
|  |  | ≥70 | Referent |  | Referent | |  | |
|  | MSI status | |  | 0.0001 |  | | <0.0001 ^d^ | |
|  |  | Non-MSI-high | Referent |  | Referent | |  | |
|  |  | MSI-high | 1.45 (1.20 - 1.76) |  | 1.91 (1.52 - 2.39) | |  | |
|  | LINE-1 methylation level | |  | 0.18 |  | | 0.0069 ^d^ | |
|  |  | ≤55 | 0.85 (0.68 - 1.05) |  | 0.75 (0.58 – 0.97) | |  | |
|  |  | 55-65 | 1.06 (0.91 - 1.24) |  | 1.15 (0.98 - 1.35) | |  | |
|  |  | >65 | Referent |  | Referent | |  | |
|  | *KRAS* mutation | |  | <0.0001 |  | | <0.0001 ^d^ | |
|  |  | Wild type | Referent |  | Referent | |  | |
|  |  | Mutated | 0.65 (0.56 - 0.74) |  | 0.57 (0.49 - 0.66) | |  | |
|  | *BRAF* mutation | |  | 0.11 |  | | <0.0001 ^d^ | |
|  |  | Wild type | Referent |  | Referent | |  | |
|  |  | Mutated | 0.86 (0.71 - 1.04) |  | 0.50 (0.40 - 0.63) | |  | |
|  | *PIK3CA* mutation | |  | 0.028 |  | | 0.0010 ^d^ | |
|  |  | Wild type | Referent |  | Referent | |  | |
|  |  | Mutated | 1.24 (1.02 - 1.50) |  | 1.41 (1.15 - 1.72) | |  | |
|  |  |  |  |  |  | |  | |
| High CD34^+^LAMB1^+^ vessel density | | | |  |  |  | |  |
|  | Age at diagnosis | |  | <0.0001 ^c^ |  | <0.0001 ^c^ | |  |
|  |  | <55 | 0.29 (0.20 - 0.41) |  | 0.28 (0.20 - 0.40) |  | |  |
|  |  | 55-69 | 0.56 (0.48 - 0.65) |  | 0.56 (0.47 - 0.65) |  | |  |
|  |  | ≥70 | Referent |  | Referent |  | |  |
|  | Body mass index | |  | <0.0001 |  | <0.0001 ^c^ | |  |
|  |  | <30 kg/m^2^ | Referent |  | Referent |  | |  |
|  |  | ≥30 kg/m^2^ | 1.80 (1.51 - 2.15) |  | 1.89 (1.58 - 2.27) |  | |  |
|  | MSI status | |  | 0.0090 |  | 0.011 ^c^ | |  |
|  |  | Non-MSI-high | Referent |  | Referent |  | |  |
|  |  | MSI-high | 1.31 (1.07 - 1.60) |  | 1.31 (1.04 - 1.65) |  | |  |
|  |  | |  |  |  |  | |  |
|  |  | |  |  |  |  | |  |
|  | *BRAF* mutation | |  | 0.36 |  | 0.012 ^c^ | |  |
|  |  | Wild type | Referent |  | Referent |  | |  |
|  |  | Mutated | 0.90 (0.73 - 1.12) |  | 0.74 (0.58 - 0.95) |  | |  |

^a^ Inverse probability weighting method was applied to reduce bias due to the data availability after cancer diagnosis.

^b^ The multivariable logistic regression model initially included age at diagnosis, birth year, sex, body mass index at diagnosis, pack-year of smoking before diagnosis, family history of colorectal cancer, MSI status, CIMP status, LINE-1 methylation level, *KRAS* mutation, *BRAF* mutation, and *PIK3CA* mutation.

^c^ A backward elimination with a threshold *P* of 0.1 was used to select variables for the final model. Only variables that remained in the final model are shown in this table.

^d^ *P*_trend_ was calculated with raw age value (year) as a continuous variable in the logistic regression model.

Abbreviations: CI, confidence interval CIMP, CpG island methylator phenotype; LINE-1, long interspersed nucleotide element-1; MSI, microsatellite instability. **Supplementary Table S8.** Distribution of cases by dichotomized age at diagnosis and vessel density categories.

| Variable | | Age at diagnosis | | |
| --- | --- | --- | --- | --- |
|  |  | <55 | 55-70 | ≥70 |
|  |  | n=52 | n=400 | n=391 |
| Overall CD34^+^ vessel density | |  |  |  |
|  | Low (n=422) | 31 (60%) | 206 (52%) | 185 (47%) |
|  | High (n=421) | 21 (40%) | 194 (48%) | 206 (53%) |
|  | |  |  |  |
| Micro CD34^+^ vessel density | |  |  |  |
|  | Low (n=422) | 26 (50%) | 205 (51%) | 191 (49%) |
|  | High (n=421) | 26 (50%) | 195 (49%) | 200 (51%) |
|  | |  |  |  |
| CD34^+^LAMB1^+^ vessel density | |  |  |  |
|  | Negative/low (n=608) | 43 (83%) | 305 (76%) | 260 (66%) |
|  | High (n=235) | 9 (17%) | 95 (24%) | 131 (34%) |

**Supplementary Table S9.** Logistic regression analysis to assess the association of age at diagnosis (predictor) with high vessel densities (binary outcome variables) without inverse probability weighting.

| Variables | | | Univariable-unadjusted | *P* value | Multivariable-adjusted | *P* value |
| --- | --- | --- | --- | --- | --- | --- |
|  |  |  | odds ratio (95% CI) |  | odds ratio (95% CI) ^a,b^ |  |
| High overall CD34^+^ vessel density | | | |  |  |  |
|  | Age at diagnosis | |  | 0.016 ^c^ |  | 0.021 ^c^ |
|  |  | <55 | 0.61 (0.34 - 1.10) |  | 0.64 (0.35 - 1.15) |  |
|  |  | 55-69 | 0.85 (0.64 - 1.12) |  | 0.86 (0.65 - 1.14) |  |
|  |  | ≥70 | Referent |  | Referent |  |
|  |  |  |  |  |  |  |
| High micro CD34^+^ vessel density | | | |  |  |  |
|  | Age at diagnosis | |  | 0.21 ^c^ |  | 0.32 ^c^ |
|  |  | <55 | 0.96 (0.54 - 1.70) |  | 1.01 (0.57 - 1.81) |  |
|  |  | 55-69 | 0.91 (0.69 - 1.20) |  | 0.93 (0.70 - 1.24) |  |
|  |  | ≥70 | Referent |  | Referent |  |
|  |  |  |  |  |  |  |
|  |  |  |  |  |  |  |
| High CD34^+^LAMB1^+^ vessel density | | | |  |  |  |
|  | Age at diagnosis | |  | 0.0002 ^c^ |  | 0.0001 ^c^ |
|  |  | <55 | 0.42 (0.20 - 0.88) |  | 0.41 (0.19 - 0.87) |  |
|  |  | 55-69 | 0.62 (0.46 - 0.85) |  | 0.61 (0.44 - 0.83) |  |
|  |  | ≥70 | Referent |  | Referent |  |

^a^ The multivariable logistic regression model initially included age at diagnosis, birth year, sex, body mass index at diagnosis, pack-year of smoking before diagnosis, family history of colorectal cancer, MSI status, CIMP status, LINE-1 methylation level, *KRAS* mutation, *BRAF* mutation, and *PIK3CA* mutation.

^b^ A backward elimination with a threshold *P* of 0.1 was used to select variables for each final model.

^c^ *P*_trend_ was calculated with raw age value (year) as a continuous variable in the logistic regression model.

Abbreviations: CI, confidence interval.

**Supplementary Table S10.** Clinical, pathological, and molecular characteristics of colorectal cancer cases according to overall CD34^+^ vessel density.

|  |  |  | Overall CD34^+^ vessel density | | | |  |
| --- | --- | --- | --- | --- | --- | --- | --- |
| Characteristics ^a^ | | All cases | Q1 | Q2 | Q3 | Q4 | *P* value ^b^ |
|  |  | N=843 | N=211 | N=211 | N=211 | N=210 |  |
| Sex | |  |  |  |  |  | 0.24 |
|  | Female (NHS) | 468 (56%) | 121 (59%) | 125 (59%) | 109 (52%) | 113 (54%) |  |
|  | Male (HPFS) | 375 (44%) | 90 (41%) | 86 (41%) | 102 (48%) | 97 (46%) |  |
| Body mass index at diagnosis (kg/m^2^) | | 26 (24-29) | 26 (24 - 29) | 25 (23 - 28) | 26 (24 - 29) | 26 (24 - 29) | 0.69 |
| Prediagnostic physical activity (MET h/week) | | 13 (3.7-29) | 11 (4.2-28) | 12 (3.1-33) | 14 (4.8-33) | 13 (3.4-26) | 0.14 |
| Prediagnostic pack-year of smoking | | 13 (3.7-29) | 10 (0 - 32) | 4 (0 - 25) | 9.5 (0 - 33) | 6 (0 - 29) | 0.91 |
| Prediagnostic alcohol (g/day) | | 7.0 (0-30) | 2.6 (0-11) | 2.0 (0-11) | 2.7 (0-14) | 2.0 (0-12) | 0.40 |
| Family history of colorectal cancer in a first-degree relative | | | | | | 2.3 (0-12) | 0.82 |
|  | Absent | 659 (79%) | 163 (78%) | 172 (83%) | 162 (78%) | 162 (78%) |  |
|  | Present | 176 (21%) | 47 (22%) | 37 (17%) | 47 (22%) | 45 (22%) |  |
| Tumor location | |  |  |  |  |  | 0.430 |
|  | Proximal colon | 420 (50%) | 100 (48%) | 109 (52%) | 104 (50%) | 107 (51%) |  |
|  | Distal colon | 248 (30%) | 65 (31%) | 54 (26%) | 64 (31%) | 65 (31%) |  |
|  | Rectum | 172 (20%) | 45 (21%) | 48 (23%) | 41 (19%) | 38 (18%) |  |
| AJCC disease stage | |  |  |  |  |  | <0.0001 |
|  | I | 183 (23%) | 30 (17%) | 36 (18%) | 58 (31%) | 59 (31%) |  |
|  | II | 254 (33%) | 64 (35%) | 66 (33%) | 56 (29%) | 68 (35%) |  |
|  | III | 226 (29%) | 64 (35%) | 61 (31%) | 53 (28%) | 48 (25%) |  |
|  | IV | 119 (15%) | 43 (24%) | 36 (18%) | 23 (12%) | 17 (9%) |  |
| Tumor differentiation | | |  |  |  |  | 0.20 |
|  | Well/ Moderate | 773 (92%) | 186 (89%) | 194 (92%) | 202 (96%) | 191 (91%) |  |
|  | Poor | 69 (8%) | 24 (11%) | 17 (8%) | 9 (4%) | 19 (9%) |  |
| MSI status | |  |  |  |  |  | 0.037 |
|  | Non-MSI-high | 674 (82%) | 175 (85%) | 172 (84%) | 171 (83%) | 156 (77%) |  |
|  | MSI-high | 143 (18%) | 31 (15%) | 31 (16%) | 35 (17%) | 46 (23%) |  |
| CIMP status | |  |  |  |  |  | 0.49 ^c^ |
|  | Negative/Low | 606 (78%) | 156 (78%) | 156 (78%) | 156 (82%) | 138 (73%) |  |
|  | High | 173 (22%) | 43 (22%) | 44 (22%) | 35 (18%) | 51 (27%) |  |
| LINE-1 methylation level | | |  |  |  |  | 0.022 ^d^ |
|  | ≤55 | 183 (22%) | 49 (23%) | 53 (25%) | 46 (22%) | 35 (17%) |  |
|  | 55-65 | 331 (39%) | 84 (40%) | 79 (37%) | 85 (40%) | 83 (40%) |  |
|  | >65 | 329 (39%) | 78 (37%) | 79 (37%) | 80 (38%) | 92 (44%) |  |
| *KRAS* mutation | |  |  |  |  |  | 0.0068 |
|  | Wild type | 490 (60%) | 117 (57%) | 109 (56%) | 123 (60%) | 141 (69%) |  |
|  | Mutant | 328 (40%) | 88 (43%) | 94 (44%) | 82 (40%) | 64 (31%) |  |
| *BRAF* mutation | |  |  |  |  |  | 0.51 |
|  | Wild type | 692 (84%) | 168 (82%) | 171 (83%) | 183 (88%) | 170 (83%) |  |
|  | Mutant | 132 (16%) | 37 (18%) | 35 (17%) | 25 (12%) | 35 (17%) |  |
| *PIK3CA* mutation | | |  |  |  |  | 0.65 |
|  | Wild type | 641 (83%) | 162 (83%) | 162 (84%) | 155 (82%) | 162 (85%) |  |
|  | Mutant | 127 (17%) | 33 (17%) | 32 (16%) | 33 (18%) | 29 (15%) |  |
| Tumor-infiltrating lymphocytes | | | |  |  |  | 0.021 |
|  | Absent/ Low | 606 (72%) | 160 (78%) | 153 (73%) | 150 (71%) | 143 (68%) |  |
|  | Intermediate | 132 (16%) | 27 (13%) | 33 (16%) | 39 (19%) | 33 (16%) |  |
|  | High | 97 (12%) | 19 (9%) | 23 (11%) | 21 (10%) | 34 (16%) |  |
|  | | | |  |  |  |  |
|  | | | |  |  |  |  |
|  | | | |  |  |  |  |
| Intratumoral periglandular reaction | | | |  |  |  | 0.0005 |
|  | Absent/ Low | 110 (13%) | 35 (17%) | 31 (15%) | 26 (13%) | 18 (9%) |  |
|  | Intermediate | 622 (74%) | 153 (74%) | 156 (74%) | 158 (75%) | 155 (74%) |  |
|  | High | 104 (12%) | 19 (9%) | 22 (11%) | 26 (13%) | 37 (18%) |  |
| Peritumoral lymphocytic reaction | | | |  |  |  | 0.0009 |
|  | Absent/ Low | 128 (15%) | 37 (18%) | 34 (16%) | 31 (15%) | 26 (12%) |  |
|  | Intermediate | 574 (69%) | 149 (72%) | 150 (72%) | 134 (64%) | 141 (67%) |  |
|  | High | 131 (16%) | 21 (10%) | 24 (12%) | 44 (21%) | 42 (20%) |  |
| Crohn's-like lymphoid reaction | | | |  |  |  | 0.0049 |
|  | Absent/ Low | 522 (74%) | 144 (82%) | 125 (72%) | 134 (75%) | 119 (67%) |  |
|  | Intermediate | 126 (18%) | 21 (12%) | 37 (21%) | 30 (17%) | 38 (21%) |  |
|  | High | 56 (8%) | 11 (6%) | 11 (6%) | 14 (8%) | 20 (11%) |  |

^a^ Percentage indicates the proportion of patients with a specific clinical, pathological, or molecular characteristic among all patients or in the overall CD34^+^ vessel density categories. For continuous variables, values are presented as the median and interquartile range (25th-75th percentile).

^b^ To compare categorical data and continuous data between the categories, the Spearman correlation test was performed using overall CD34^+^ vessel density as a ordinal variable.

^c^ The Spearman correlation test was performed with CIMP status as a 9-level ordinal variable.

^d^ The Spearman correlation test was performed with LINE-1 methylation level as a continuous variable.

Abbreviations: AJCC, American Joint Committee on Cancer; CIMP, CpG island methylator phenotype; HPFS, Health Professionals Follow-up Study; LINE-1, long interspersed nucleotide element-1; MET, metabolic equivalent of task; MSI, microsatellite instability; NHS, Nurses’ Health Study.**Supplementary Table S11.** Clinical, pathological, and molecular characteristics of colorectal cancer cases according to CD34^+^LAMB1^+^ vessel density.

|  |  |  | CD34^+^LAMB1^+^ vessel density | | |  |
| --- | --- | --- | --- | --- | --- | --- |
| Characteristics ^a^ | | All cases | Negative | Low | High | *P* value ^b^ |
|  |  | N=843 | N=372 | N=236 | N=235 |  |
| Sex | |  |  |  |  | 0.056 |
|  | Female (NHS) | 468 (56%) | 219 (59%) | 129 (55%) | 120 (51%) |  |
|  | Male (HPFS) | 375 (44%) | 153 (41%) | 107 (45%) | 115 (49%) |  |
| Body mass index at diagnosis (kg/m^2^) | | 26 (24-29) | 26 (23 - 29) | 25 (23 - 28) | 26 (24 - 30) | 0.26 |
| Prediagnostic physical activity (MET h/week) | | 13 (3.7-29) | 12 (4-28) | 12 (3.4-29) | 14 (3.8-30) | 0.90 |
| Prediagnostic pack-year of smoking | | 7.0 (0-30) | 8.0 (0 - 32) | 5.5 (0 - 31) | 8.5 (0 - 27) | 0.82 |
| Prediagnostic alcohol (g/day) | | 2.3 (0-12) | 2.5 (0-11) | 2.0 (0-13) | 2.2 (0-11) | 0.69 |
| Family history of colorectal cancer in a first-degree relative | | | | |  | 0.81 |
|  | Absent | 659 (79%) | 290 (79%) | 184 (79%) | 185 (79%) |  |
|  | Present | 176 (21%) | 79 (21%) | 49 (21%) | 48 (21%) |  |
| Tumor location | |  |  |  |  | 0.37 |
|  | Proximal colon | 420 (50%) | 179 (48%) | 120 (51%) | 121 (52%) |  |
|  | Distal colon | 248 (30%) | 115 (31%) | 64 (27%) | 69 (30%) |  |
|  | Rectum | 172 (20%) | 77 (21%) | 52 (22%) | 43 (18%) |  |
| AJCC disease stage | |  |  |  |  | 0.24 |
|  | I | 183 (23%) | 85 (24%) | 44 (20%) | 54 (25%) |  |
|  | II | 254 (33%) | 118 (34%) | 68 (31%) | 68 (31%) |  |
|  | III | 226 (29%) | 105 (30%) | 64 (29%) | 57 (26%) |  |
|  | IV | 119 (15%) | 39 (11%) | 42 (19%) | 38 (18%) |  |
| Tumor differentiation | |  |  |  |  | 0.78 |
|  | Well/ Moderate | 773 (92%) | 340 (91%) | 218 (92%) | 215 (92%) |  |
|  | Poor | 69 (8%) | 32 (9%) | 18 (8%) | 19 (8%) |  |
| MSI status | |  |  |  |  | 0.99 |
|  | Non-MSI-high | 674 (82%) | 293 (82%) | 198 (86%) | 183 (81%) |  |
|  | MSI-high | 143 (18%) | 66 (18%) | 33 (14%) | 44 (19%) |  |
| CIMP status | |  |  |  |  | 0.65 ^c^ |
|  | Negative/ Low | 606 (78%) | 268 (77%) | 168 (79%) | 170 (78%) |  |
|  | High | 173 (22%) | 80 (23%) | 46 (21%) | 47 (22%) |  |
| LINE-1 methylation level | |  |  |  |  | 0.88 ^d^ |
|  | ≤55 | 183 (22%) | 82 (22%) | 48 (20%) | 53 (23%) |  |
|  | 55-66 | 331 (39%) | 141 (38%) | 99 (42%) | 91 (39%) |  |
|  | >65 | 329 (39%) | 149 (40%) | 89 (38%) | 91 (39%) |  |
| *KRAS* mutation | |  |  |  |  | 0.43 |
|  | Wild type | 490 (60%) | 222 (62%) | 135 (58%) | 133 (59%) |  |
|  | Mutant | 328 (40%) | 138 (38%) | 97 (42%) | 93 (41%) |  |
| *BRAF* mutation | |  |  |  |  | 0.050 |
|  | Wild type | 692 (84%) | 294 (81%) | 201 (87%) | 197 (86%) |  |
|  | Mutant | 132 (16%) | 70 (19%) | 30 (13%) | 32 (14%) |  |
| *PIK3CA* mutation | |  |  |  |  | 0.10 |
|  | Wild type | 641 (83%) | 279 (81%) | 181 (86%) | 181 (85%) |  |
|  | Mutant | 127 (17%) | 66 (19%) | 29 (14%) | 32 (15%) |  |
| Tumor-infiltrating lymphocytes | | |  |  |  | 0.88 |
|  | Absent/ Low | 606 (72%) | 270 (73%) | 168 (71%) | 168 (71%) |  |
|  | Intermediate | 132 (16%) | 53 (14%) | 36 (15%) | 43 (18%) |  |
|  | High | 97 (12%) | 46 (12%) | 28 (12%) | 23 (10%) |  |
|  | | |  |  |  |  |
|  | | |  |  |  |  |
|  | | |  |  |  |  |
| Intratumoral periglandular reaction | | |  |  |  | 0.65 |
|  | Absent/ Low | 110 (13%) | 54 (15%) | 31 (13%) | 25 (11%) |  |
|  | Intermediate | 622 (74%) | 265 (72%) | 176 (76%) | 181 (77%) |  |
|  | High | 104 (12%) | 50 (13%) | 26 (11%) | 28 (12%) |  |
| Peritumoral lymphocytic reaction | | |  |  |  | 0.52 |
|  | Absent/ Low | 128 (15%) | 56 (15%) | 39 (17%) | 33 (14%) |  |
|  | Intermediate | 574 (69%) | 247 (67%) | 163 (70%) | 164 (71%) |  |
|  | High | 131 (16%) | 66 (18%) | 30 (13%) | 35 (15%) |  |
| Crohn's-like lymphoid reaction | | |  |  |  | 0.13 |
|  | Absent/ Low | 522 (74%) | 242 (75%) | 156 (79%) | 124 (67%) |  |
|  | Intermediate | 126 (18%) | 53 (17%) | 29 (15%) | 44 (24%) |  |
|  | High | 56 (8%) | 26 (8%) | 13 (6%) | 17 (9%) |  |

^a^ Percentage indicates the proportion of patients with a specific clinical, pathological, or molecular characteristic among all patients or in the CD34^+^LAMB1^+^ vessel density categories. For continuous variables, values are presented as the median and interquartile range (25th-75th percentile).

^b^ To compare categorical data and continuous data between the categories, the Spearman correlation test was performed using CD34^+^LAMB1^+^ vessel density as a ordinal variable.

^c^ The Spearman correlation test was performed with CIMP status as a 9-level ordinal variable.

^d^ The Spearman correlation test was performed with LINE-1 methylation level as a continuous variable.

Abbreviations: AJCC, American Joint Committee on Cancer; CIMP, CpG island methylator phenotype; HPFS, Health Professionals Follow-up Study; LINE-1, long interspersed nucleotide element-1; MET, metabolic equivalent of task; MSI, microsatellite instability; NHS, Nurses’ Health Study.

^
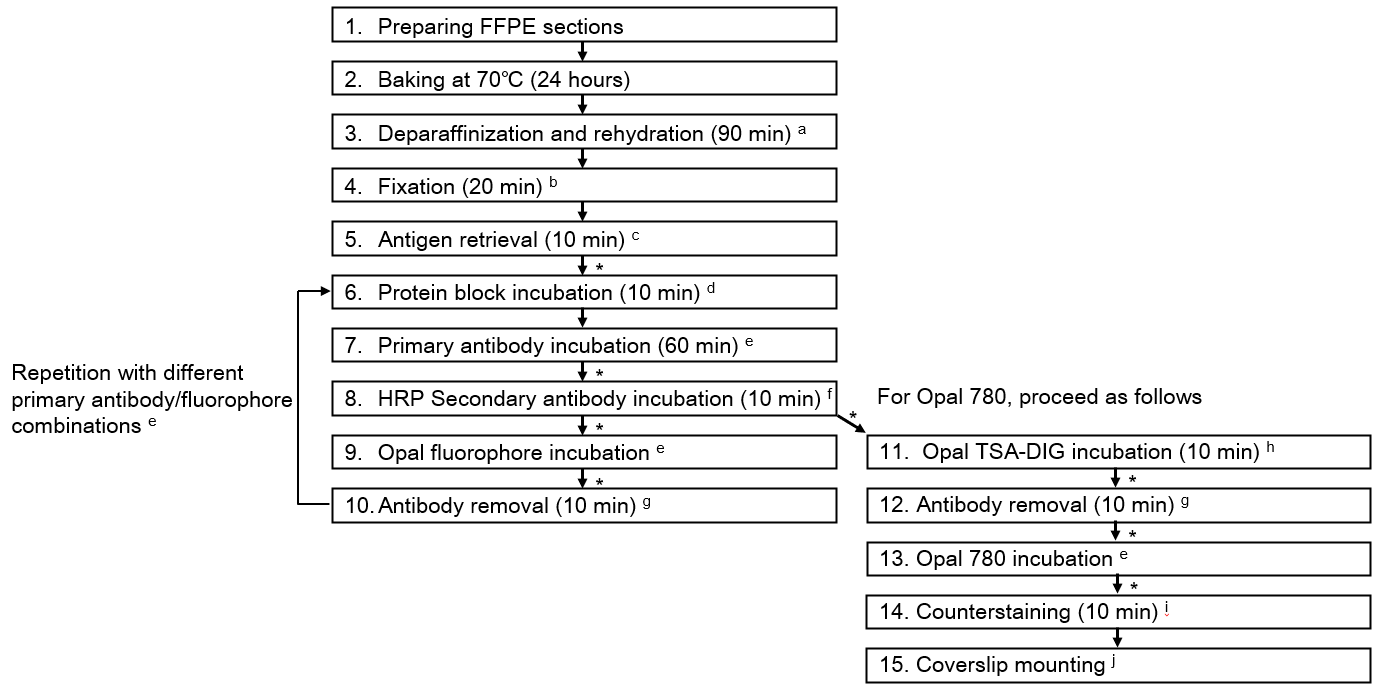
^

**Supplementary Figure S1.** Flowchart of the multiplex immunofluorescence. FFPE, formalin-fixed paraffin-embedded.

^a^ Deparaffinization: Xylene (X3P-1GAL, Fisher Scientific, Pittsburgh, PA, USA), 5 times for 10 minutes. Dehydration: 100% ethanol (HC-800-1GAL, Fisher Scientific), 3 times for 10 minutes, 95% ethanol for 5 minutes, and 70% ethanol for 5 minutes.

^b^ With 10% formalin (SF98-20, Fisher Scientific).

^c^ With Microwave oven (NE-1054F, Panasonic, Osaka, Japan) in AR6 buffer (AR600250ML, Akoya Biosciences, Hopkinton, MA, USA).

^d^ Antibody diluent/block (ARD1001EA, Akoya Biosciences).

^e^ See Supplementary Table S1 for details about antibodies and fluorophores.

^f^ Opal Polymer HRP Ms + Rb (ARH1001EA, Akoya Biosciences).

^g^ With Microwave oven (NE-1054F, Panasonic) in AR9 buffer (AR900250ML, Akoya Biosciences).

^h^ Opal TSA-DIG (Opal 780 reagent pack, FP1501001KT, Akoya Biosciences) with 1:100 dilution.

^i^ Spectral DAPI (FI1490, Akoya Biosciences).

^j^ Epredia Signature Series Cover Glass (12450S, Fisher Scientific)); ProLong Diamond Antifade Mountant (P36970, Thermo Fisher Scientific, Waltham, MA, USA).

* Wash with TBS-T (TBS [sc-24951, Santa Cruz Biotechnology, Dallas, TX, USA], Tween 20 [9005-64-5, Millipore Sigma, Burlington, MA, USA]), 3 times for 2 minutes.


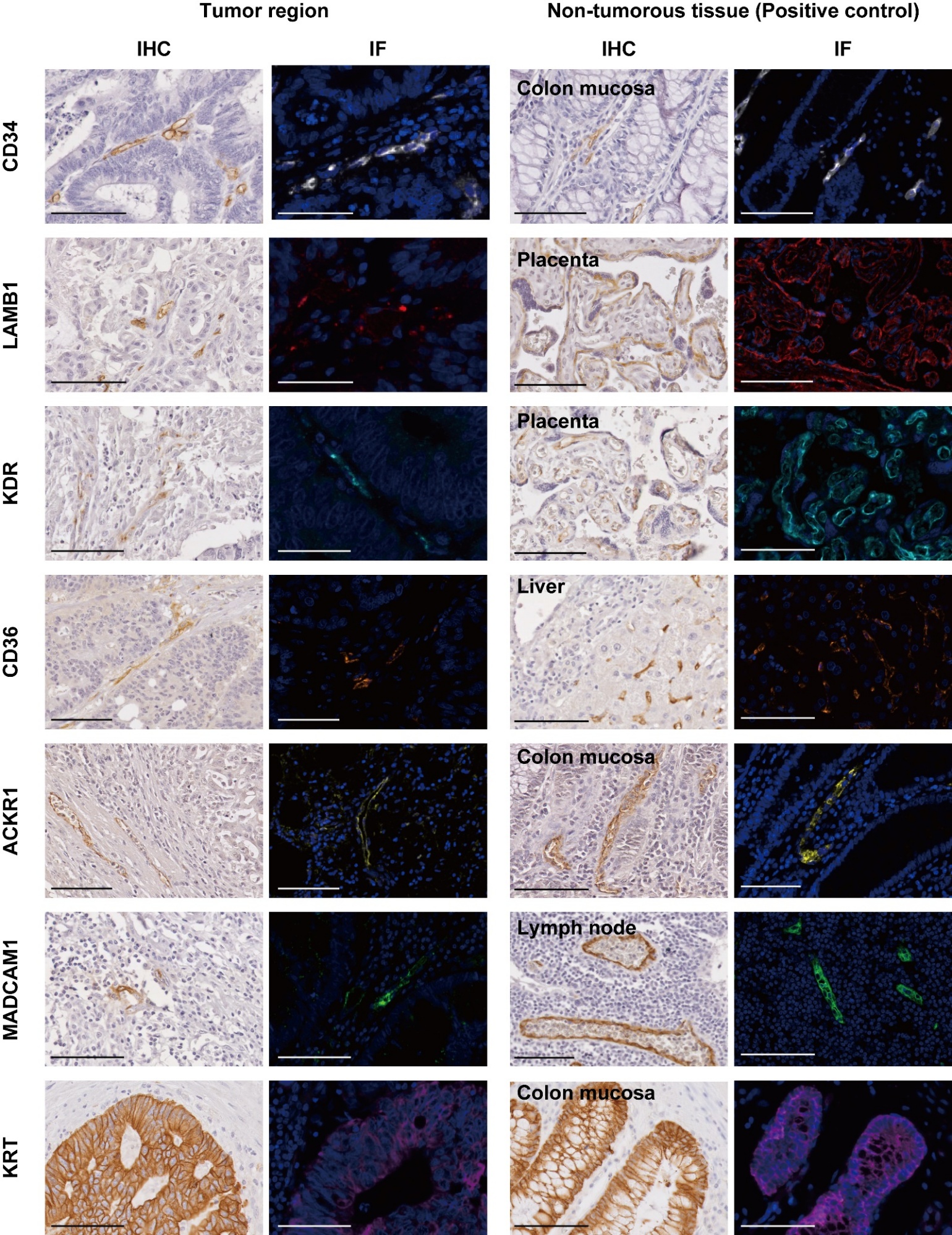


**Supplementary Figure S2.** Comparison of staining patterns between immunohistochemistry (IHC) and immunofluorescence (IF). Scale bar: 100 μm.


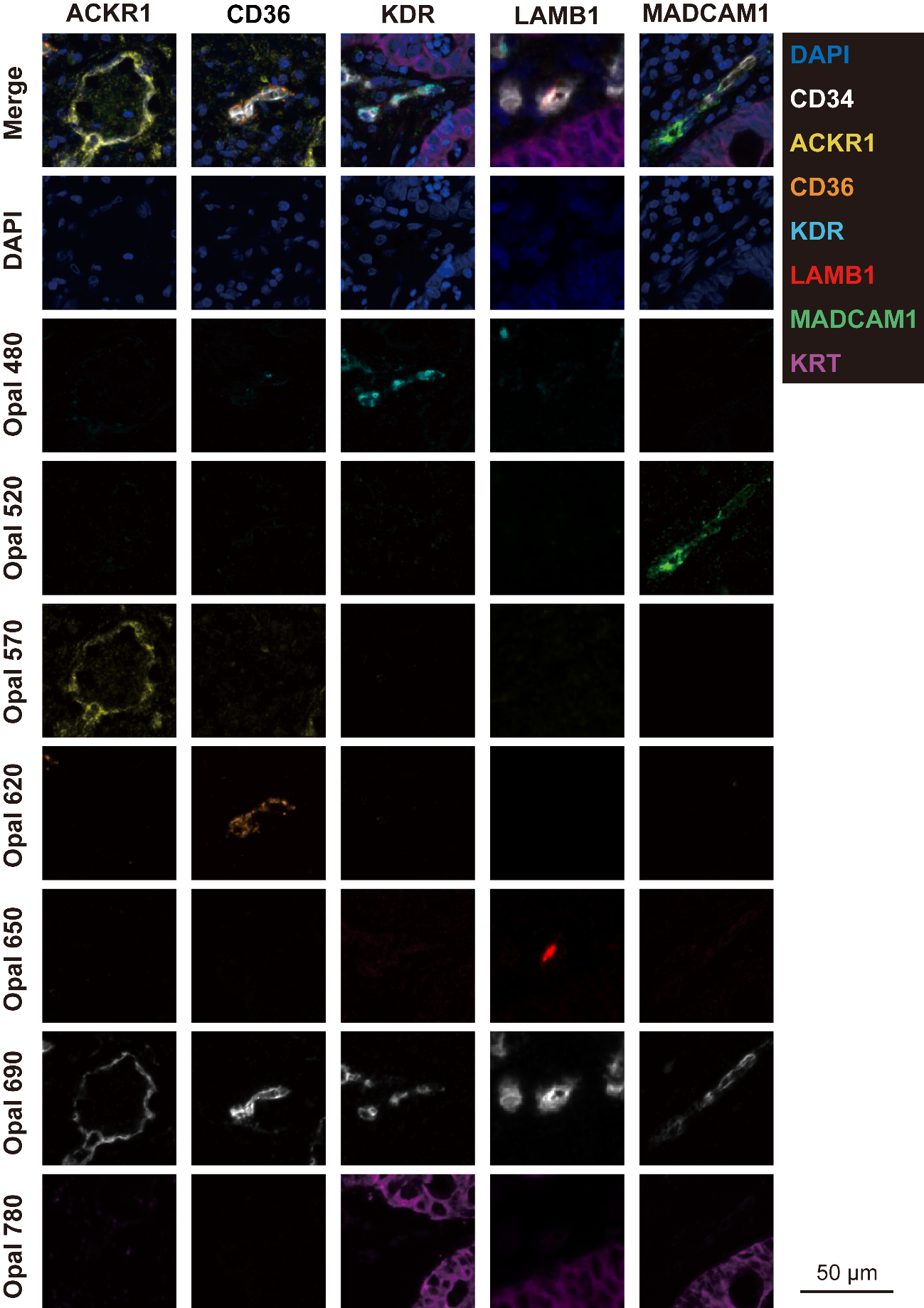


**Supplementary Figure S3.** Multiplex immunofluorescent images of vessels positive for each endothelial cell marker in colorectal cancer. The images of each channel confirm that fluorescence leakage is well controlled.


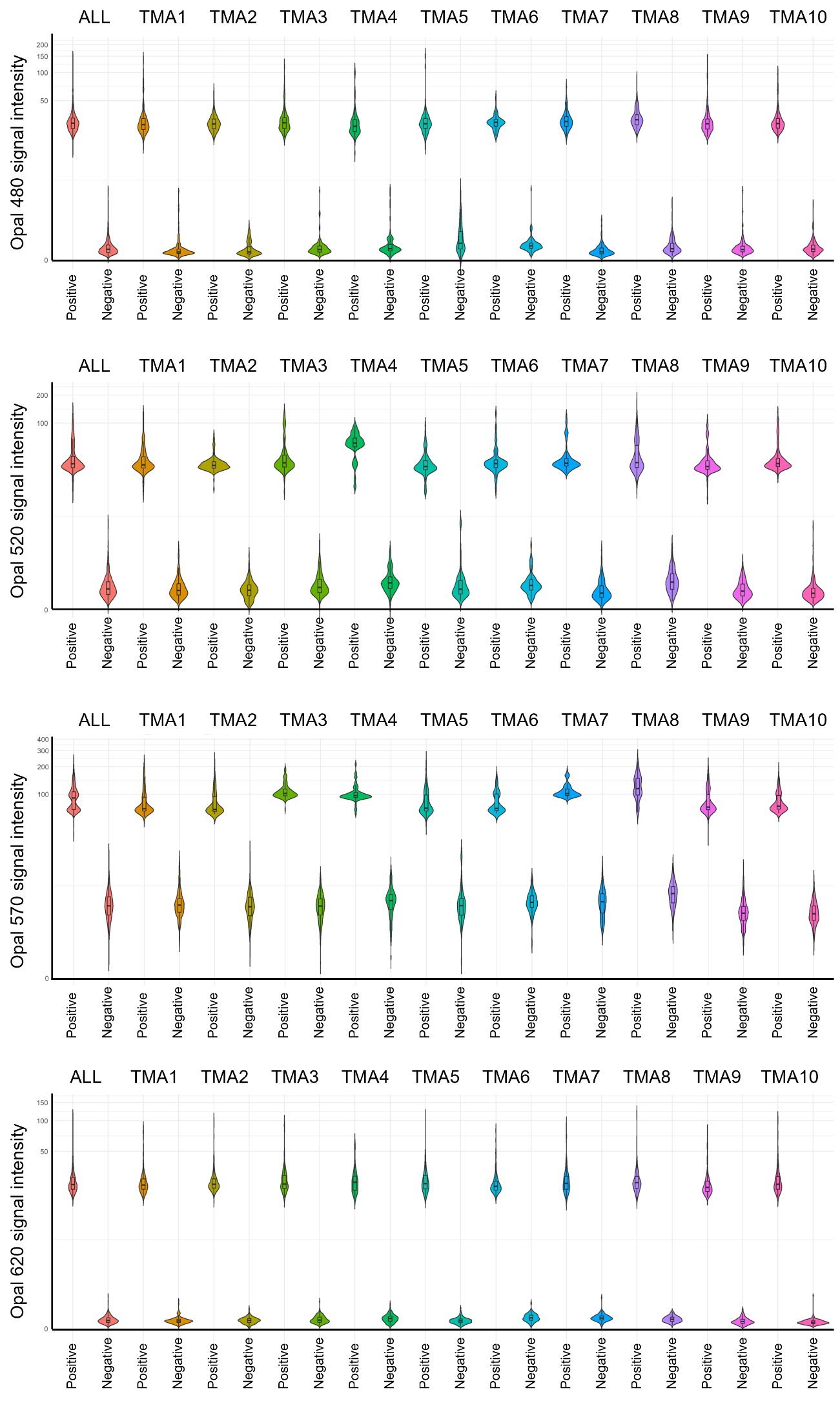


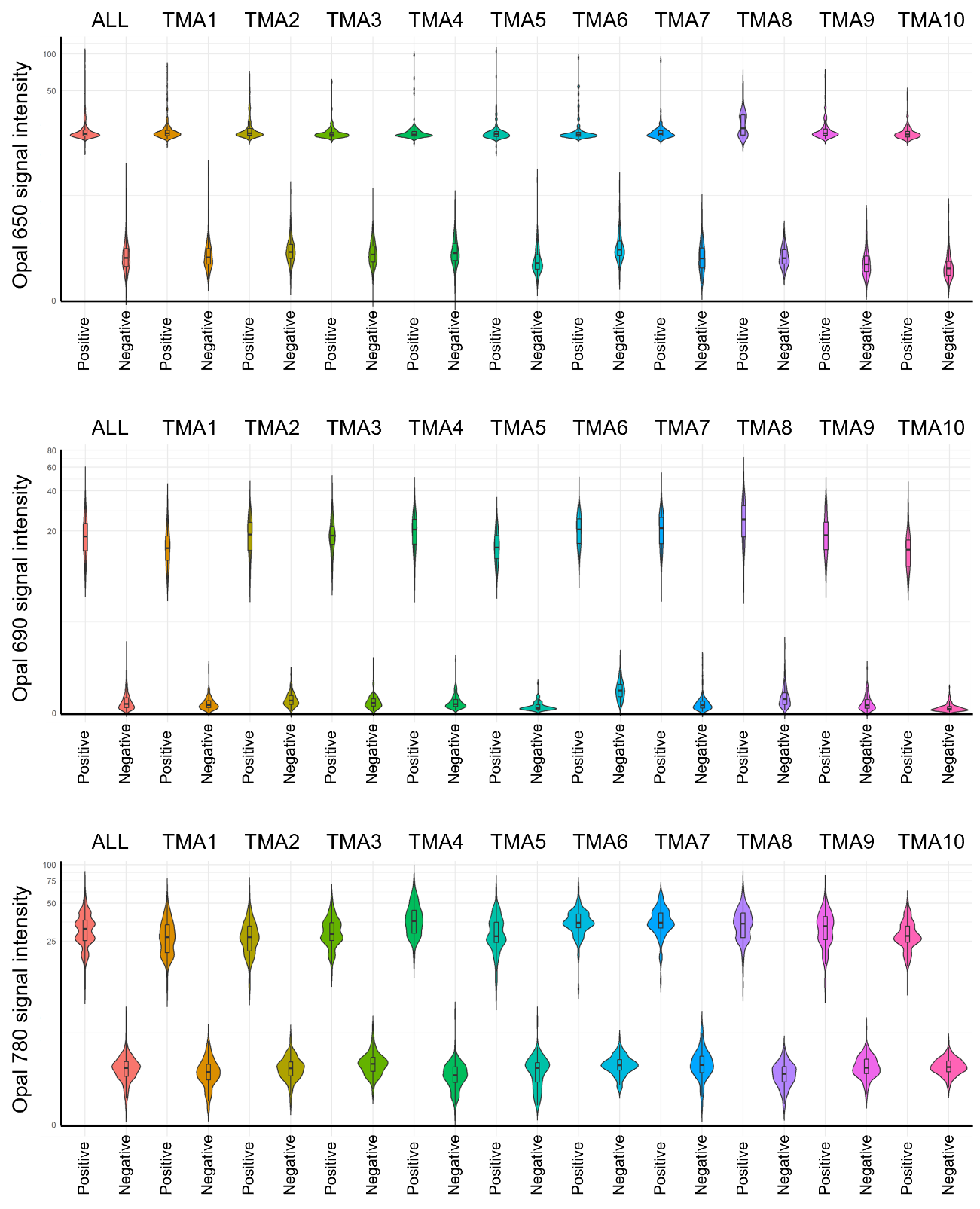


**Supplementary Figure S4.** Signal intensity on each tissue microarray (TMA) slide. Grayscaled immunofluorescent images were binarized (Positive/ Negative) at the pixel level by Otsu’s method based on its signal intensity. The mean signal intensities within the Positive and Negative areas were calculated for each core.


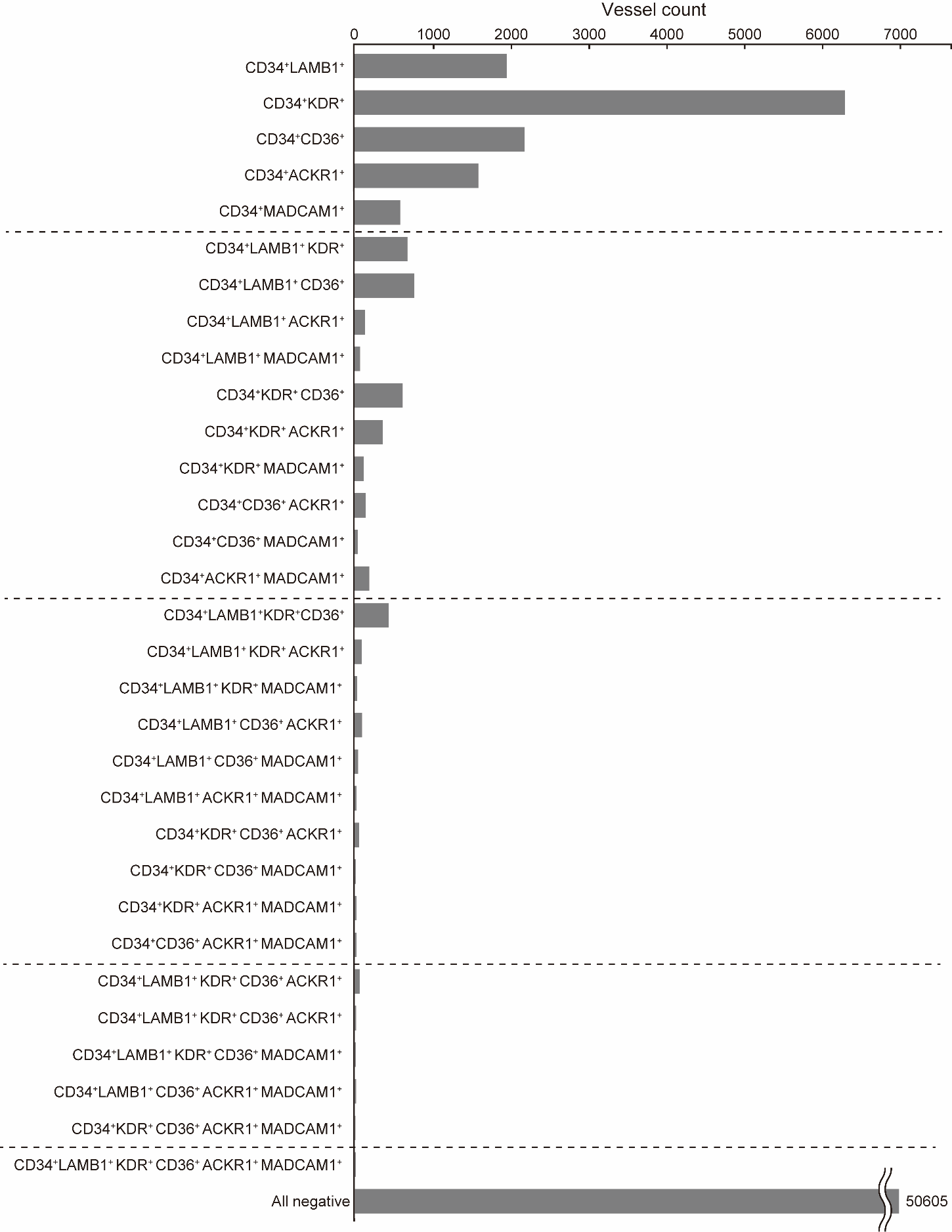


**Supplementary Figure S5.** Details of each endothelial cell marker expression in CD34^+^ vessels.


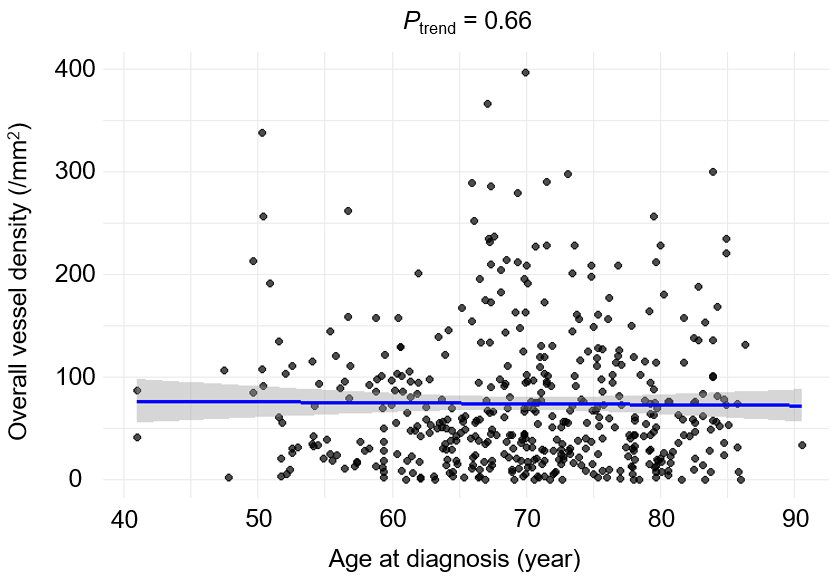


**Supplementary Figure S6.** Scatter plot showing the relationship between age at diagnosis and overall CD34^+^ vessel density in non-tumorous mucosa. Among the tissue microarray cores derived from the resected specimens used in this study, cores containing lamina propria were analyzed (N=287). The blue line indicates the linear regression line, and the gray shaded area represents the 95% confidence interval.

The Spearman's rank correlation test was performed on continuous data to calculate *P_trend_*.
